# Comparative Time-Scale Gene Expression Analysis Highlights the Infection Processes of Two *Amoebophrya* Strains

**DOI:** 10.3389/fmicb.2018.02251

**Published:** 2018-10-02

**Authors:** Sarah Farhat, Isabelle Florent, Benjamin Noel, Ehsan Kayal, Corinne Da Silva, Estelle Bigeard, Adriana Alberti, Karine Labadie, Erwan Corre, Jean-Marc Aury, Stephane Rombauts, Patrick Wincker, Laure Guillou, Betina M. Porcel

**Affiliations:** ^1^Génomique Métabolique, Genoscope, Institut François Jacob, CEA, CNRS, Univ. Evry, Université Paris-Saclay, Evry, France; ^2^Communication Molecules and Adaptation of Microorganisms, National Museum of Natural History, CNRS, Paris, France; ^3^Genoscope, Institut François Jacob, CEA, Evry, France; ^4^Sorbonne Universités, Université Pierre et Marie Curie-Paris 6, CNRS, UMR 7144, Station Biologique de Roscoff, Roscoff, France; ^5^Department of Plant Biotechnology and Bioinformatics, Ghent University, Ghent, Belgium

**Keywords:** amoebophrya, syndiniales, parasite, gene expression, infection, oxidative stress response, plankton, dinoflagellates

## Abstract

Understanding factors that generate, maintain, and constrain host-parasite associations is of major interest to biologists. Although little studied, many extremely virulent micro-eukaryotic parasites infecting microalgae have been reported in the marine plankton. This is the case for *Amoebophrya*, a diverse and highly widespread group of Syndiniales infecting and potentially controlling dinoflagellate populations. Here, we analyzed the time-scale gene expression of a complete infection cycle of two *Amoebophrya* strains infecting the same host (the dinoflagellate *Scrippsiella acuminata*), but diverging by their host range (one infecting a single host, the other infecting more than one species). Over two-thirds of genes showed two-fold differences in expression between at least two sampled stages of the *Amoebophrya* life cycle. Genes related to carbohydrate metabolism as well as signaling pathways involving proteases and transporters were overexpressed during the free-living stage of the parasitoid. Once inside the host, all genes related to transcription and translation pathways were actively expressed, suggesting the rapid and extensive protein translation needed following host-cell invasion. Finally, genes related to cellular division and components of the flagellum organization were overexpressed during the sporont stage. In order to gain a deeper understanding of the biological basis of the host-parasitoid interaction, we screened proteins involved in host-cell recognition, invasion, and protection against host-defense identified in model apicomplexan parasites. Very few of the genes encoding critical components of the parasitic lifestyle of apicomplexans could be unambiguously identified as highly expressed in *Amoebophrya*. Genes related to the oxidative stress response were identified as highly expressed in both parasitoid strains. Among them, the correlated expression of superoxide dismutase/ascorbate peroxidase in the specialist parasite was consistent with previous studies on *Perkinsus marinus* defense. However, this defense process could not be identified in the generalist *Amoebophrya* strain, suggesting the establishment of different strategies for parasite protection related to host specificity.

## Introduction

Parasitism is probably one of the most widespread lifestyles in nature. The intrinsic relationship established between a parasite and its host implies a delicate and unstable balance, where the parasite subverts the metabolism of the host to fulfill its needs while the host develops strategies allowing it to prevent and survive infection. The large majority of host-parasite models studied to date have been selected for ecological or medical relevance. Indeed, while most studies are aimed at plant and animal parasites (Torchin and Mitchell, 2004; Hart, 2011; Tellier and Brown, 2011; Kutzer and Armitage, 2016), not much is known about parasites of marine microbes, especially those infecting phytoplanktonic micro-eukaryotes.

One example of this overlooked group corresponds to organisms belonging to the *Amoebophrya* spp. genus (MALV or Marine Alveolates), a diverse clade of parasitoids infecting dinoflagellates, some of them responsible of toxic algal blooms (Cachon, 1964; Park et al., 2004). Syndinean parasites are basal to dinoflagellates, a sister group to apicomplexans such as *Plasmodium falciparum* and *Toxoplasma gondii*. Molecular barcoding has revealed the wide diversity and occurrence of *Amoebophrya* spp. in all marine planktonic ecosystems (Guillou et al., 2008; de Vargas et al., 2015). Nevertheless, these marine alveolates remain a poorly known group when compared to other members of the marine plankton. The high-quality ribosomal RNA database SILVA encompasses today over 2,000 SSU sequences corresponding to *Amoebophrya* species: only 39 SSU sequences have been described today, labeled as “*Amoebophrya* sp.,” with more than 2,000 SSU sequences still accounting for uncultured species (Glöckner et al., 2017).

The concepts of specialist and generalist parasite have been extensively surveyed. Parasite specialization relies on multiple constraints, including the life-history traits and ecology of the host, as well as co-evolutionary processes guiding host-parasite interactions. The specificity of a parasite for a particular host is constrained by evolutionary processes underlying key steps during the infection, such as penetration and the takeover of the host metabolism, as well as the ability of the parasite to extract resources from the host it cannot acquire in any other way. Coastal planktonic ecosystems are characterized by strong environmental changes and rapid turnovers (Dia et al., 2014; Blanquart et al., 2016). While parasites with narrow niches (e.g., the specialists) are presumably favored during periods of environmental stability, those with broader niches (e.g., the generalists) are likely favored during environmental instability or heterogeneity (Kassen, 2002). Consequently, the majority of parasites of marine microalgae should theoretically be generalists. In fact, many documented micro-eukaryotic parasites infecting dinoflagellates are generalists (Garcés et al., 2013a,b; Lepelletier et al., 2014a,b). Members of the *Amoebophrya* clade constitute a clear detour from this hypothesis, where some strains display various degrees of host specialization (Coats and Park, 2002) resulting in the occurrence of a large number of specialist parasites among the group (Chambouvet et al., 2008). Host range specificity is a constantly evolving capacity driven by the molecular mechanisms underlying the host-parasite relationship.

*Amoebophrya* is a parasite that cannot survive for long outside its host. A complete life-cycle lasts between 3 and 5 days in our culture conditions, depending upon the identity of both the host and the parasite. While completing its life-cycle, *Amoebophrya* alternates between a free-swimming flagellated infective stage, so-called dinospore, rapidly followed by a uninuclear (trophont) and multinuclear (sporont) growth phase within its host (Miller et al., 2012). After attachment to the surface, the parasitoid invades the host cell, with some strains infesting and digesting the host's nucleus first before sporulation, while others remains in the cytoplasm (Coats and Park, 2002). The parasite, now a trophont, starts an active feeding. After increasing in size, the parasite starts a series of nuclear divisions and flagellar replications while following feeding. The mature trophont evolves into a multicellular sporont so-called the “beehive,” due to its similarity with a honey beehive. This sporont is released into the water as a vermiform and then separates into hundreds of dinospores. Those flagellated dinospores can survive around 3–15 days while searching for a new host to infect (Cachon, 1964; Coats, 1999).

Several studies have characterized the morphological and physiological changes taking place during infection by Amoebophrya (Park et al., 2002; Miller et al., 2012; Kim and Park, 2016). Still, the deep changes observed throughout the *Amoebophrya* life cycle suggest the requirement of different sets of parasite proteins so as to contact, interact and survive within the host cell. Yet, to date, only little information is available on the molecular processes and the parasite effectors responsible of this parasitoid-host interaction at a gene expression level (Bachvaroff et al., 2009; Lu et al., 2014).

Many aspects of the host-parasite interplay remain unsolved. For instance, how does the infection affect the parasite life cycle inside the host at a molecular level? Which are the genes and pathways triggered throughout the infection? And how does *Amoebophrya* gene expression change between the free-living stage and its life inward the host? Here, we present a comparative study, at a transcriptomic level, of the infective cycle of two distinct *Amoebophrya* strains (one specialist and one more generalist) in the same host *Scrippsiella acuminata*. These *Amoebophrya* strains belong to MALVII clade 2 (Guillou et al., 2008, http://www.aquasymbio.fr/) and share 99.96% of nucleotide similarity in their SSU rDNA gene sequence. Both strains were isolated from the Penzé estuary (North-West of France, English Channel) where this parasite cluster was observed to infect the host *Scrippsiella* spp. (Chambouvet et al., 2008). Accordingly, in culture, the two strains infect *S. acuminata* (STR2), but whilst A25 is able to infect only this host, the strain A120 was also able to infect other co-occurring species isolated from the Penzé (*S. donghaenis, Scrippsiella trochoidea* STR2 and another genus (*Heterocapsa triquetra*). We monitored changes in the expression of *Amoebophrya* genes throughout the infection in order to better understand the mechanisms underlying invasion and resistance to host defenses.

## Materials and methods

### Origin of the host and the *Amoebophrya* A25 and A120 parasitic strains

Strains of hosts and parasites come from the Penzé estuary (North-West of France, English Channel, 48°37′N; 3°56′W). A culture of the non-toxic dinoflagellate *Scrippsiella acuminata* [previously known as *S. trochoidea*, (Kretschmann et al., 2015)] was previously established from the germination of a single cyst collected in 2005 from sediment. A culture of the non-toxic dinoflagellates *Heterocapsa triquetra* was established after isolation of a single vegetative cell using a glass micropipette from water collected the July 6th 2007 at salinity 27‰. A monoclonal strain was subsequently obtained for each taxon after the re-isolation of a single vegetative cell (names of strains: ST147 for *S. acuminata* and HT150 for *H. triquetra*). From natural samples, *Amoebophrya*-like parasites infecting dinoflagellates were detected and isolated thanks to their natural bright green autofluorescence using an epifluorescence microscope (BX51, Olympus) equipped with the U-MWB2 cube [450- to 480-nm excitation, 500-nm emission, (Coats and Bockstahler, 1994)]. A single infected *Scrippsiella* sp. and a single infected *H. triquetra*, in late-stage of infection, were picked from samples collected on the 15th of June 2009 and the 13th of June 2011, respectively and incubated into exponentially growing *S. acuminata* (ST147) and *H. triquetra* (HT150), respectively. These two *Amoebophrya*-like strains were then sequentially re-isolated six times for parasites in ST147 and 6 times for parasites in HT150. These two strains have been called A25 (specialist parasitoid, primary host ST147) and A120 (generalist parasitoid, primary host HT150). After a series of cycles in their primary hosts, the strain maintained in *H. triquetra* was successfully transferred and maintained in *S. acuminata* (ST147).

All strains have been deposited at the Roscoff Culture Collection, http://roscoff-culture-collection.org/, with the following numbers: ST147 = RCC1627, HT150 = RCC3596, A25 = RCC4383, A120 = RCC4398).

### Acquisition of *Amoebophrya* transcriptomes over an infection cycle

In order to analyze the transcriptional profiles of the two *Amoebophrya* strains during infection, dinospores were collected once (3 replicates for A120 and 2 replicates for A25) by a gentle filtration through a 0.6-μm polycarbonate filters (Whatman) using a vacuum pump. Also, gene expression of both parasites was monitored through the full infection cycle including intracellular stages. Host cells were prepared using 1 L ventilated flasks and pooled prior to the experiment. Parasite cells were prepared in 600 mL ventilated flasks. Freshly harvested dinospores were separated from the remaining host cells by a gravity filtration using 5-μm polycarbonate filter (Whatman) and pooled. We initiated incubation by transferring 150 mL of the dinospore culture and 300 mL of exponentially growing host culture (ST147) into a 600 ml culture flask (one per replicate and time). Aliquots of 1.5 mL of culture were fixed with glutaraldehyde (0.25% final concentration) for at least 15 min and stored at −80°C before analysis. Cell concentrations were determined by flow cytometry (FACSAria, Becton Dickinson) after DNA staining with SYBR Green-I at a final dilution of 1/50,000. Dinospores:hosts ratio were thus estimated a posteriori to be 30x and 10x for A120 and A25, respectively (Table S1B). Cultures of host cells were sampled in triplicate every 6 h including the T0 (host alone, no parasite) for 36 h for A120 and 44 h for A25. We obtained 7 time steps (21 samples in total) and 9 time steps (27 samples in total) for A120 and A25, respectively. Cells were collected by a gentle filtration through a 10-μm polycarbonate filters (Whatman) using a vacuum pump. Filters were immediately submerged into 4 mL of TriZol, carefully mixed during 1–2 min and flash-frozen in liquid nitrogen. Samples were stored at −80°C until extraction.

The prevalence of infection was also estimated for each time point of the infection (Figure S1, Table S1). Four stages were reported. The first corresponds to all hosts with at least one parasite in the cytoplasm, the second corresponds to all hosts with at least one parasite in the nucleus, the third corresponds to all hosts with digested nucleus (intermediate infection) and for the fourth, almost all the host is digested (mature trophont).

### Transcriptome sequencing

Total RNA was purified directly from filters using Direct-zol RNA Miniprep kit (ZymoResearch, Irvine, CA) following manufacturer's protocol. First, the tube containing the filter immersed in TriZol was incubated 10 min at 65°C. Then, after addition of an equal volume of EtOH 100% and vortexing, the mixture was loaded into a Zymo-SpinIIC column and centrifuged for 1 min at 12,000 g. The loading and centrifugation steps were repeated until exhaustion of the mixture. RNA purification was completed by prewash and wash steps following the manufacturer's instructions and RNA was directly eluted in 45 μL nuclease-free water. The in-column DNAse step was replaced by a more efficient post-extraction DNAse treatment using Turbo DNA-free kit (Thermo Fisher Scientific, Waltham, MA), according to the manufacturer's rigorous DNase treatment procedure. After two rounds of 30 min incubation at 37°C, the reaction mixture was purified with the RNA Clean and Concentrator-5 kit (ZymoResearch) following the procedure described for retention of > 17 nt RNA fragments. Total RNA was quantified with RNA-specific fluorimetric quantitation on a Qubit 2.0 Fluorometer using Qubit RNA HS Assay (ThermoFisher Scientific). RNA quality was assessed by capillary electrophoresis on an Agilent Bioanalyzer, using the RNA 6000 Pico LabChip kit (Agilent Technologies, Santa Clara, CA).

RNA-Seq library preparations were carried out from 1 to 4 μg total RNA using the TruSeq Stranded mRNA kit (Illumina, San Diego, CA) and the manufacturer's protocol, which allows mRNA strand orientation (sequence reads occur in the same orientation as anti-sense RNA). In short, poly(A)+ RNA was selected with oligo(dT) beads, chemically fragmented and converted into single-stranded cDNA using random hexamer priming. Then, the second strand was generated to create double-stranded cDNA. Strand specificity was achieved by quenching the second strand during final amplification thanks to incorporation of dUTP instead of dTTP during second strand synthesis. Then, ready-to-sequence Illumina library was quantified by qPCR using the KAPA Library Quantification Kit for Illumina Libraries (KapaBiosystems, Wilmington, MA), and libraries profiles evaluated with an Agilent 2100 Bioanalyzer (Agilent Technologies). Each library was sequenced using 101 bp paired end reads chemistry on a HiSeq2000 Illumina sequencer.

**Figure 1:**
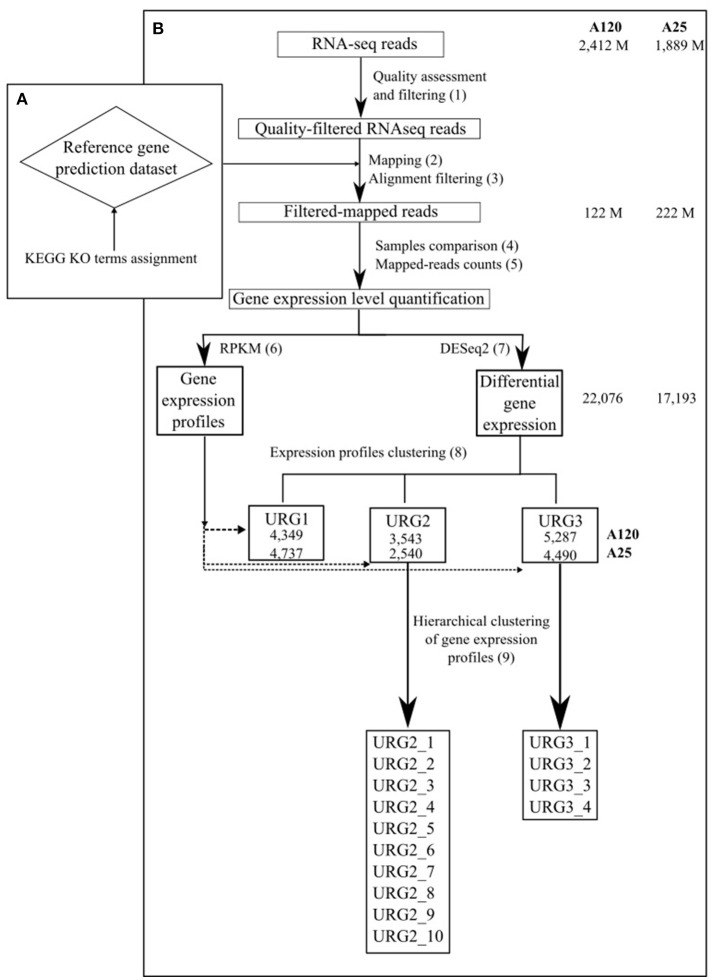
Schematic outline of the gene expression analysis approach used in the study. The total number of RNA-Seq reads, number of reads in high-quality alignements on the gene prediction dataset of the corresponding *Amoebophrya* strain, number of DEGs and number of genes in each URG in A120 and A25 respectively are shown.

The sequencing generated 48 reads files corresponding to 7 time points for *Amoebophrya* A120 and 9 time points for *Amoebophrya* A25, with 3 replicates for each time point. Also, five read files with only dinospore stage were generated for each organism (2 replicates for A120 and 3 for A25). The raw data was filtered to remove any clusters with too much intensity corresponding to bases other than the called base. RNA-Seq reads were cleaned in a three-step procedure (Figure 1, step 1): (i) sequencing adapters and low-quality nucleotides (quality value < 20) were removed, (ii) sequences between the second unknown nucleotide (N) and the end of the read were removed, (iii) reads shorter than 30 nucleotides after trimming were discarded, together with reads and their mates mapping onto run quality control sequences (PhiX genome). These trimming steps were achieved using fastx_clean (http://www.genoscope.cns.fr/fastxtend), a home-made software based on the FASTX toolkit (http://hannonlab.cshl.edu/fastx_toolkit/). Moreover, ribosomal RNA-like reads were excluded using SortMeRNA (Kopylova et al., 2012). The number of cleaned RNA sequences obtained is shown in Table S2.

### Gene expression analysis

Complete genomes of both *Amoebophrya* A120 and A25 strains, have been recently sequenced, assembled and annotated using a combination of deep coverage Nanopore and Illumina sequencing approaches (unpublished). The cleaned *Amoebophrya* A120 and A25 RNA-Seq reads were aligned against the transcripts inferred from the *Amoebophrya* genome annotation (26,427 genes for A120; 28,076 for A25) of their respective strains using BWA-MEM (Li and Durbin, 2010) with default parameters for each time point. While no significant match could be seen at T0-point (host alone), around 90% (A120) and 88% (A25) of the *Amoebophrya* dinospore reads were aligned on the A120 and A25 inferred transcripts, respectively. This procedure allowed distinguishing between the reads from the parasite and the host. Then, the resulting mapping was filtered using 95% identity and 85% of predicted gene coverage as criteria (Figure 1, step 2 and 3; Table S2, Figure S3). Expression levels were quantified by counting the mapped reads for each *Amoebophrya* genes. Read counts of each triplicate samples were analyzed by comparing, in a pairwise fashion, the read counts distribution and median correlation between each pairs of replicates, together with the SERE procedure (Schulze et al., 2012) (Figure 1, step 4 and 5; Tables S2, S3). Only samples with a SERE value less than 1,7 were kept for further analysis (with the exception for the replicates of A25 dinospore). Then, the read counts (Figure 1, step 6 and 7) were used for differential gene expression (DGE) analysis using DESeq2 (Love et al., 2014) comparing all vs all time points. Genes were considered as differentially expressed using a corrected *p*-value < 1e-5 and an absolute fold change >2 as thresholds. In parallel, RPKM (Reads Per Kilobase per Millions) was calculated with the same read counts data in order to visualize gene expression profiles all along *Amoebophrya* life cycle.

### Functional repertoires of amoebophrya A120 and A25

Both *Amoebophrya* A120 and A25 translated inferred transcripts were assigned to functional categories using the Biomolecular Relations in Information Transmission and Expression (BRITE) functional hierarchies from the Kyoto Encyclopedia of Genes and Genomes (KEGG) database (Kanehisa et al., 2016) (https://www.genome.jp/kegg/brite.html) in two steps. Briefly, KEGG mapping of both *Amoebophrya* proteomes was performed using BLASTp between *Amoebophrya* A120 and A25 proteins and the proteins included in the Eukaryotes KEGG Orthology (KO) database using an *e*-value ≤ 10e-5 as cutoff (8,690 and 8,360 proteins with a least one significant match were kept for further analysis). KO assignment for each *Amoebophrya* protein was carried out by keeping the best KO, based on cumulative bit-score and the KO with a cumulative score similar to the best one (90% of the score). As a result, KO terms were assigned to 5,983 and 5,774 proteins in A120 and A25 respectively (23 and 21% of the entire A120 and A25 translated inferred transcripts dataset respectively). Finally, KO terms assigned to both *Amoebophrya* strains were classified according to BRITE functional categories (Kanehisa et al., 2016). A single gene product may be associated with multiple BRITE annotations within a single category. At last 5,856 (A120) and 5,774 (A25) proteins were related to a BRITE annotation.

### Search for the expression of specific gene families in *Amoebophrya* spp.

We investigated the molecular processes involved in infection by *Amoebophrya* spp. To do so, selected proteins involved in invasion processes from annotated proteomes of the model apicomplexan parasites *Plasmodium falciparum* (Pf) and *Toxoplasma gondii* (Tg), were used as queries for BLASTp searches (at e-value 1e-5) using the NCBI BLAST+ 2.2.28 package against the *Amoebophrya* A120 and A25 sequences. Moreover, the Best Reciprocal Hits (BRH) were identified between each *Amoebophrya* predicted proteome and Pf and Tg proteomes. The resulting candidates in *Amoebophrya* were subsequently manually validated and their identity confirmed by BLASTp against the NR database (NCBI Resource Coordinators, 2016) and phylogenetic analysis, followed by manual expertise on the selected *Amoebophrya* candidates. Moreover, orthologous relationships between both *Amoebophrya* strains for the selected target genes were defined using a BRH on pairwise alignments between the *Amoebophrya* predicted proteomes [Smith-Waterman algorithm (Codani et al., 1999) (BLOSUM62, gapo = 10, gape = 1); only the alignments with a score greater than 300 were retained].

Similarly, genes involved in the reactive oxygen species (ROS) response were identified by the same approach, using the annotated genes of the reactive oxygen species (ROS) scavenging network of *Arabidopsis thaliana* as queries (Mittler et al., 2004). Multiple alignment of ascorbate peroxidase of *Amoebophrya* A120 and A25, and ascorbate peroxidases from *Perkinsus marinus* was performed using MAFTT (Kuraku et al., 2013), followed by Boxshade for visualization (http://www.ch.embnet.org/software/BOX_form.html). A similar approach was also used for superoxide dismutase candidate sequences from *Amoebophrya* A120 and A25. Phylogenetic analysis was performed using NJ phylogeny (Kuraku et al., 2013).

### Expression profiles clustering

To distinguish different patterns of gene expression during the *Amoebophrya* life cycle, upregulated genes (URG) showing differential expression were categorized into three groups (Figure 1, step 8). The first group (URG1) is composed of genes that were upregulated when comparing the dinospore stage to every other time point of the infection following the below formula

$$\underset{i\in \{6,12,18,24,30,36\}}{\cap }D\left(T0\;vs\;Ti\right)\;\leq 0\;\cap \;\sum_{i\in \left\{6,12,18,24,30,36\right\}}D\left(T0\;vs\;Ti\right)\;\neq 0$$

where D is the log2 fold change of the DGE. Furthermore, only genes with a RPKM value > than RPKM in every other time point were kept in the URG1 group.

The second group (URG2) is composed of genes that were upregulated at the beginning of the infection (genes upregulated in at least one of the three first time points of the experiment T6, T12 and T18 when compared to all the other time points) following the below formula

$$\underset{i\in \left\{6,12,18\right\}}{\cap }D\left(T0\;vs\;Ti\right)\;\geq 0\;\cap D\left(Ti\;vs\;T24,30,36\right)\;\leq 0\;\cap$$

$$\sum_{i\in \left\{6,12,18\right\}}D\left(T0\;vs\;Ti\right)+D\left(Ti\;vs\;T24,30,36\right)\;\neq 0$$

Moreover, only genes with a RPKM value in T6, T12, T18 time-points that were bigger than RPKM values in every other time point were kept in the URG2 group.

And finally, the third group (URG3) accounted for genes that were upregulated at the end of the infection (in at least one of the last time points of the experiment, T24, T30 and T36 for A120 and T24, T30, T36, T42 and T44 for A25, when compared to all the other time points) following the below formula

$$\underset{i\in \{0,6,12,18\}}{\cap }D\left(Ti\;vs\;T24,30,36\right)\geq \;0\;\cap \;\sum_{i\in \left\{0,6,12,18\right\}}D\left(Ti\;vs\;T24,30,36\right)\;\neq \;0$$

Moreover, only genes with a RPKM values in time-points related to the end of the infection that were bigger than RPKM values in every other time point were kept in the URG3 group.

Only genes with RPKM levels higher than 4 at all time-points were kept for further analysis.

### Correlated gene expression profiles within URG groups

Members of the URG2 and URG3 groups (beginning and end of the *Amoebophrya* host cell infection) were independently clustered into different sub-groups regarding the correlation of the gene expression during the entire experiment (Figure 1, step 9). For all genes composing URG2 and URG3, the rcorr function from Hmisc package in R was computed on all expression values (RPKM) at all time points. Only expression values with a correlation with *P*-value up to 0.1 were kept. Then, an hclust clustering (R) was used on the correlation values of all these upregulated genes. A cluster was validated if the mean and the median of the correlation of the genes in the cluster was higher than 0.5. 7 and 10 sub-groups were identified in URG2 for *Amoebophrya* A120 and A25 respectively (Tables S4, S5). 4 sub-groups were identified in URG3 in both strains (Tables S6, S7).

## Results

### Global gene expression in *Amoebophrya* A120 and A25

The strategy followed is outlined in Figures 1A,B (metrics in Table S2; for details see Materials and Methods). A total of 48 samples encompassing a full *Amoebophrya* infection cycle (21 for A120 and 27 for A25) were submitted to Illumina pair-end sequencing, generating 2412 M and 1889 M sequence reads per *Amoebophrya* A120 and A25 respectively (Tables S2, S3). Cleaned sequence reads from each A120 (342 M) and A25 (160 M) samples were aligned on the gene prediction dataset of the corresponding *Amoebophrya* strain, yielding high-quality alignments for a significant fraction of the reads (222M for A120 and 122M for A25; Tables S2, S3). After discarding sequence reads from non-accurate replicates (16 samples for A120 and 21 samples for A25, Table S3; for details see Methods), read-counts were used to quantify gene expression levels (RPKM) and for pairwise differential gene expression analysis (DESeq) between each time step of the *Amoebophrya* A120 and A25 life cycles (Tables S2, S4). This final DESeq-based strategy allowed us to identify the fraction of genes differently expressed at key steps of the parasite life cycle.

**Figure 2:**
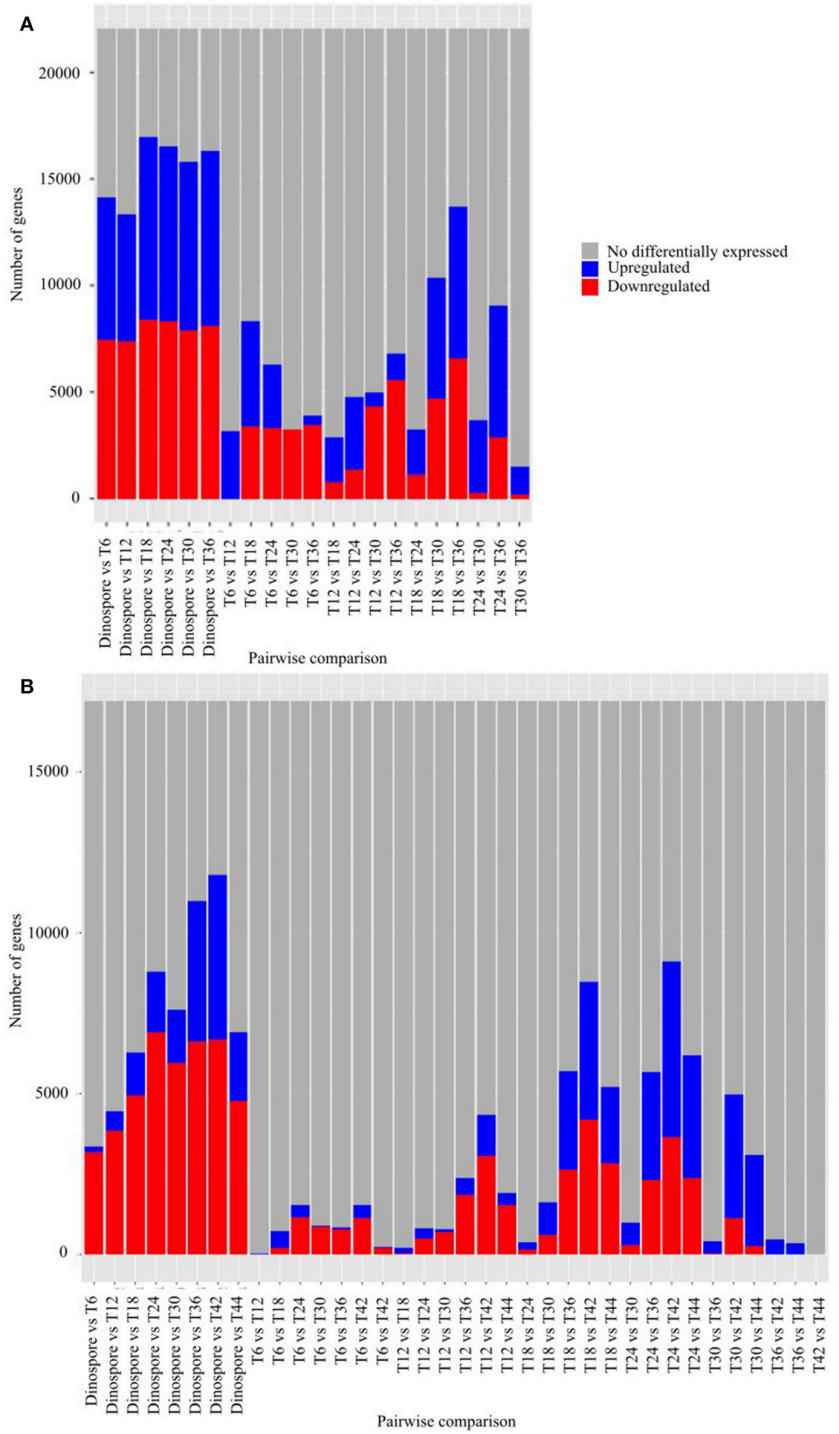
Differential gene expression is mainly observed while comparing the free-living stage to the host-infecting stages of *Amoebophrya* strains. Number of genes after pairwise comparison between gene expression of *Amoebophrya* A120 **(A)** and A25 **(B)** time points (T6, T12, and T18 corresponding to the beginning of the infection; T24, T30, and T36 in **(A)**, T24, T30, T36, T42, and T44 in **(B)** corresponding to the end of the infection) along the *Scrippsiella acuminata* infection are shown. The blues bars represent downregulated genes, the red bars represent upregulated genes and the gray bars genes not differentially expressed concerning that specific pairwise comparison.

A total of 22,076 and 17,193, corresponding to 84 and 61% of the predicted genes, were differentially expressed in at least one of the sampled stages of the *Amoebophrya* life cycle in A120 and A25, respectively (Figure 2). Surprisingly, most of the differentially expressed genes (DEGs), being up or downregulated, occurred between the free-living and the intracellular stages in both *Amoebophrya* strains with number of DEGs ranging from 13,345 to 16,940 in A120 and from 3,357 to 11,802 genes in A25 (Figure 2).

**Figure 3:**
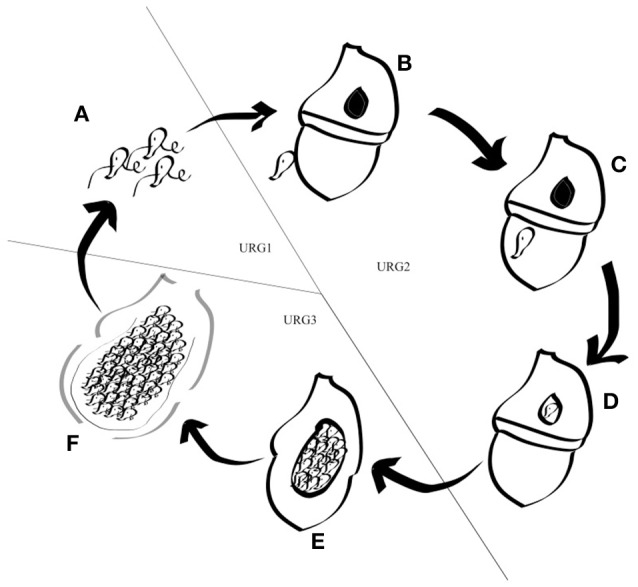
Schematic representation of the *Amoebophrya* life cycle. The different stages of the *Amoebophrya* sp. life cycle are represented as detailed in the text. The three major steps defined by significant changes in gene expression levels are shown.

**Figure 4:**
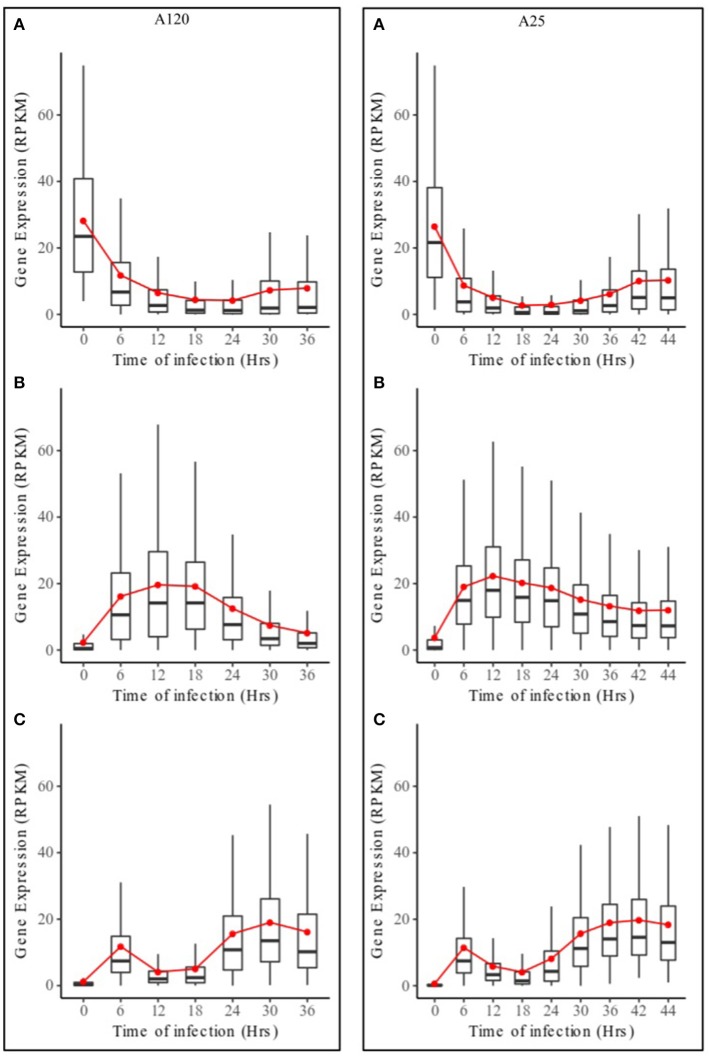
Expression profiles of URG groups in *Amoebophrya* A120 and A25. Boxplot of RPKM expression values for *Amoebophrya* A120 (left) and A25 (right) genes belonging to URG1 **(A)**, URG2 **(B)** and URG3 **(C)** at each sampled time point. The red line corresponds to the mean RPKM expression value.

Based on DESeq analysis, three upregulated gene groups - URG1, URG2, and URG3 were defined, composed of genes being overexpressed at key stages of the parasites life cycles (Figures S2, S3). Overall, 13,179 (A120) and 11,767 (A25) of the *Amoebophrya* differentially expressed genes were grouped in these three groups. URG1 comprises genes upregulated at the dinospore stage (Figure 3A, Figures S2A, S3A). Contrary to the URG1, the URG2 and URG3 groups were defined according to the prevalence and progress of *Amoebophrya*-infected hosts at each time point of the experiment (Figure S1): while URG2 encompasses genes upregulated at the beginning (T6-T12-T18, corresponding to the passage of the parasite from the cytoplasm to the nucleus of the host cell; Figures S2, S3 panel B and Figures 3B–D), URG3 contains genes upregulated at the end (T24 up to the end of the sampling, corresponding to the two last stages of the parasitoid life cycle (digested host nucleus up to fully mature trophont); Figures S2, S3C, Figures 3E,F) of the host cell infection, respectively (Figures S2B,C, S3B,C; for details see Materials and Methods). URG1 is composed of 4,349 and 4,737 genes, URG2 is composed of 3,543 and 2,540 genes and URG3 is composed of 5,287 and 4,490 genes for A120 and A25 respectively. The expression levels of genes belonging to each URG were explored all along the *Amoebophrya* life cycle by RPKM analysis. Expression profiles of the upregulated genes belonging to each of these URG groups displayed similar overall patterns between the two strains [Figures 4A (URG1), 4B (URG2), and 4C (URG3)].

**Figure 5:**
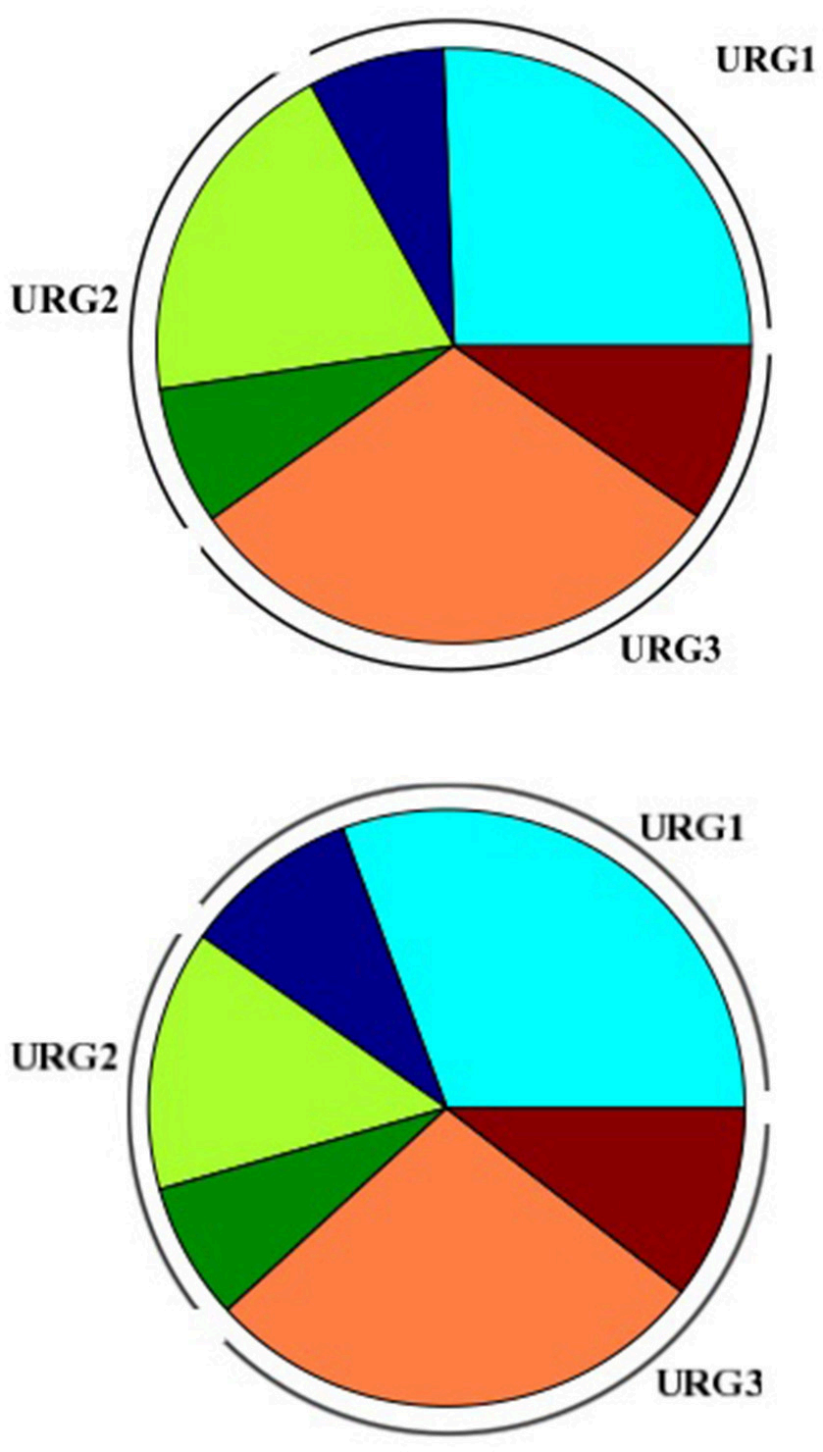
Piechart representing distribution of KO terms assignment in the Amoebophrya A120 **(up)** and A25 **(down)** URGs. URG1 (blue), URG2 (orange), and URG3 (red); no-KO term (light colors), KO-term (dark colors).

**Figure 6:**
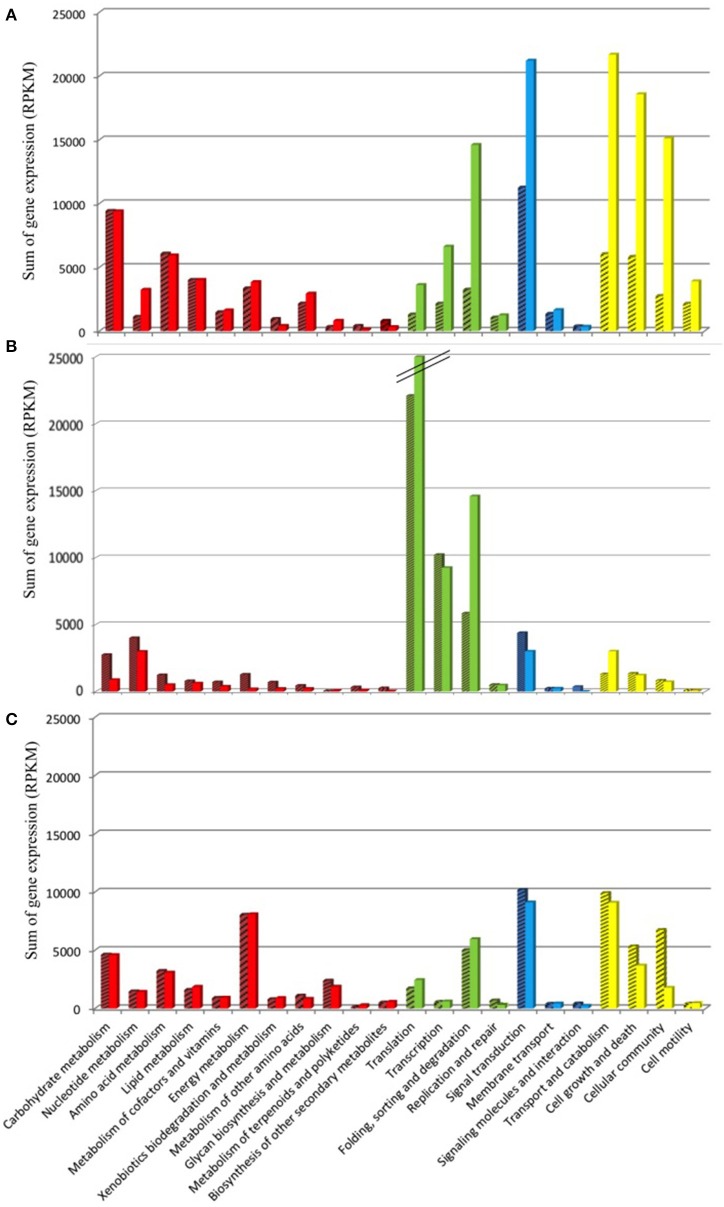
Functional KEGG categorization of genes upregulated along the *Amoebophrya* life cycle. The bar graphs correspond to the sum of the RPKM gene expression values **(A)**, dinospore stage; **(B)**, beginning of the host-infection; **(C)**, end of the host-infection, corresponding to functional BRITE-categories (level B). The main BRITE-categories used (level A) are shown in different colors: Metabolism (red), Genetic information processing (green), Environmental information processing (blue) and Cellular processes (yellow). *Amoebophrya* A120 (dashed bars) and A25 (dotted bars).

The KEGG database was used as resource in an attempt to link the variation in *Amoebophrya* gene expression with the biology of the parasite. RNA-Seq reads, corresponding to 42% (93M, A120) and 47% (57M, A25) of the total reads mapping to the *Amoebophrya* A120 and A25 predicted proteome, were aligned to *Amoebophrya* genes with a KO term. Genes with no KO term are amongst the most highly expressed. Overall, only 24% (A120) and 27% (A25) of the upregulated genes had KO term assignments. A similar distribution of KO terms was observed among the three URG categories, with most of the upregulated genes (77, 72, and 76% for A120 URG1, URG2, and URG3; 76, 63, and 72% for A25 URG1, URG2, and URG3) lacking KO term assignment (Figure 5). Figure 6 illustrates the expression level of functional BRITE categories along the *Amoebophrya* A120 and A25 life cycles. We have chosen to quantify the magnitude of the expression signal for a given BRITE category as the sum of the RPKM values of all genes assigned to the category, and this for each of the URGs previously defined (URG1, free-living; URG2, beginning and URG3, end of the infection). In the next steps of this analysis, we focused solely on genes associated to a KO term in each of the URG groups.

### *Amoebophrya* A120 and A25 genes upregulated at the free-living dinospore stage

A total of 979 (A120) and 1,127 (A25) of the genes upregulated at the free-living dinospore stage (URG1) had a BRITE category (Figure 6A, Figure S4A, Table S5). Most of these URG1 genes fell into the “Cellular Processes” category in both strains, with the “Signal transduction” and “Folding, sorting and degradation” categories (“Environmental Information Processing”) also being overrepresented. Moreover, URG1 genes related to “Metabolism”-BRITE categories were as well overexpressed in dinospores, with expression levels stretching from 9,412 to 1,112 and 9,377 to 3,228 for A120 and A25 respectively (Figure 6A).

Genes likely encoding for actin beta/gamma 1 [*e*-value of 2E-126 for A120 and 0 for A25; RPKM of 781 (A120) and 638 (A25)] and ATP-dependent RNA helicase DDX5/DBP2 (*e*-value of 9E-128 for A120 and 9e-180 for A25] with a max expression of 961.5 (A120) and 552 (A25) RPKM) and the L-lactate dehydrogenase (cytochrome) [EC:1.1.2.3] [e-value of 9e-91 and 9e-87; max expression values of 465.75 and 799.47 for A120 and A25, respectively] were among the most 10-upregulated genes during the dinospore stage in both strains.

A large variety of proteases were also identified in the URG1 group of both strains: Lon-like ATP-dependent proteases, cathepsins F, C, D and X and ATG4 cysteine proteases, together with candidates of the ubiquitin-mediated proteolysis pathway were highly upregulated, with expression values ranging from 60 to 209 (A120) and from 58 to 276 (A25) RPKM.

Moreover, genes encoding for proteins related to the “Cytoskeleton proteins” category were also upregulated in the free-living stage of the parasite at a lower extent (Figure S4), with dynein (sum of expression of 47 and 170 RPKM for A120 and A25 respectively) and kinesin family members [total expression level of 498 (A120) and 789.6 RPKM for A25] among others.

Among the URG1 group, 190 and 206 genes were upregulated at the free-living stage but displayed no differential expression pattern during the intracellular stages for A120 and A25, respectively (Figure S5). Some of the most overexpressed genes include those related to the serine/threonine-protein phosphatase PP1 catalytic subunit (total RPKM 332 for A120 end 436 for A25), ABC transporters (with a total expression level of 243 for A120 and 568 for A25) and ATP-binding cassette KO terms in both strains.

Protein-coding genes for the essential metabolic pathways needed for the parasite survival could be found overexpressed at the dinospore stage (Figure S5). Indeed, the most highly expressed KEGG-annotated genes in this dinospore-specific upregulated genes group code for enzymes involved in the nucleotide, lipid [e.g., A120 12-oxophytodienoic acid reductase [EC:1.3.1.42] with an expression of 224 RPKM, and methylsterol monooxygenase [EC:1.14.13.72] for A25, maximal expression value of 252 RPKM as examples] and carbohydrate [e.g., A120 GDP-L-fucose synthase [EC:1.1.1.271] with an expression of 333 RPKM, and phosphoenolpyruvate carboxykinase (ATP) [EC:4.1.1.49] for A25 with an expression of 311 RPKM].

Most of the genes in the dinospore-specific URGs were attributed to the “Carbohydrate metabolism” category for both A120 and A25 strains. In particular, 8 and 7 genes encode for the endoglucanase, proteins implicated in the starch and sucrose metabolism, with expression values varying from 3 to 43 RPKM (A120) and from 9 to 196 RPKM (A25).

Differences were also identified in the upregulated genes for the dinospore stage between the two strains. URG1 genes of the generalist A120 were related to processes such as endocytosis [e.g., cytohesin (1E-22 *e*-value), with a maximal expression of 530 RPKM vs. a maximal of 3 RPKM for A25 at dinospore stage], energy production [creatine kinase (9E-71 *e*-value), with a maximal expression of 935 RPKM in A120 vs. a maximal of 332 RPKM for A25 at dinospore stage] and active transporters [like the sodium-coupled neutral amino acid transporter belonging to the solute carrier family (9,00E-11 *e*-value) with a maximal expression of 575 RPKM vs. a maximal value of 26 for A25 at dinospore stage]. In the specialist strain A25, upregulated genes of URG1 encoded for tubulin beta (0 as *e*-value): 1,622 RPKM vs. 2,611 RPKM for A120 at dinospore stage and alpha: 8,496 RPKM vs. 3,587 for A120 at dinospore stage), heat shock 70 kDa protein 1/8 (*e*-value of 0; 1,952 RPKM against 795 RPKM for A120) and the molecular chaperone HtpG (*e*-value of 0; 1,934 RPKM against 413 RPKM for A120). Surprisingly, genes coding for proteins of the intraflagellar transport (IFT) complex A and B (73 and 11 RPKM, respectively) were also overexpressed in the A25 dinospore stage (URG1).

The aspartate aminotransferase, involved in the tropane, piperidine and pyridine alkaloid biosynthesis, is one of the most expressed gene (73 RPKM) for A120 among the dinospore-specific genes, together with the aspartate beta-hydroxylase [EC:1.14.11.16] enzyme (381 RPKM). Still, these enzymes were not found in the same DEG profile in A25. On the other hand, three genes coding for cellulose 1,4-beta-cellobiosidase [EC:3.2.1.91] were highly expressed (206 RPKM) at URG1 only in A120. In fact, no orthologous gene was found expressed in A25.

### Parasite genes triggered at the beginning of the infection

Only 28% of upregulated genes had BRITE category annotation during the first steps of the host-cell infection (URG2). The URGs from this group mainly corresponded to the Genetic information processing BRITE category (Figure 6B, Figure S4B). Interestingly, these URGs showed higher levels of expression when compared to URG1 (dinospore) and URG3 (end of host infection) (Figure 6B). In particular, genes related to the translation and transcription pathways (239 and 285 genes in *Amoebophrya* A120 and A25, respectively) were the most overrepresented in URG2 among the BRITE functional units (Figure 6B, Figure S4B). Amongst those were putative genes coding for proteins of ribosomal subunits, translation initiation factors [eIF1, eiF2, eIF3, eIF4 (A, B, G), and eIF5B], elongation factors and RNA transport (Table S5). In addition, several putative protease-coding genes, including UFM1- and sentrin-specific proteases and serine-type peptidases, were identified as upregulated during the beginning of the parasitoid infection (Figure S4B).

The genes belonging to URG2 were clustered into seven (A120) and ten (A25) sub-groups (Table S6) according to correlated expression levels all along the parasite's life cycle (for details see Materials and Methods). Among those, three sub-groups in each species are enriched in one or few functional categories. In fact, A120 URG2_1 (with a maximum expression at 12 h post-infection, mean RPKM value of 30), A120 URG2_2 (with an expression peak at 18 h post-infection, mean RPKM value of 19) in A120, and A25 URG2_1 (with a maximum of expression at 12 h post-infection, mean RPKM value of 28) and A25 URG2_5 (with a peak of expression at 12 h post-infection, mean RPKM value of 22) in A25 had an overrepresentation of genes involved in translation and transcription such as initiation factors, spliceosome and ribosome biogenesis. Another interesting functional sub-group (URG2_6 in A120 and URG2_5 in A25) were composed of genes involved in folding, sorting and degradation, such as exosome, cytoskeleton and transporters proteins families.

### Upregulated parasite genes at the end of infection

The majority of the highly expressed genes by the end of the host infection (URG3) were mainly assigned to BRITE categories such as cellular processes [transport and catabolism (129 and 115 genes for A120 and A25 respectively], cellular community (49 and 36 genes for A120 and A25 respectively) and cell growth and death (79 and 60 genes for A120 and A25 respectively), and processes related to signal transduction (177 and 181 genes for A120 and A25 respectively) and energy metabolism (62 and 64 genes for A120 and A25 respectively) (Figure 6C). For instance, we identified several genes encoding kinase-related proteins and cyclins, directly involved in mitosis, to be highly expressed in the last stage of the infection. Components of the flagellum organization, such as kinesin, dyneins and the intraflagellar transport (IFT) complex, were also significantly overrepresented at this late stage of the parasite's life cycle (Table S5). Interestingly, we found that genes related to the energy metabolism displayed higher levels of expression in the URG3 compared to both the dinospore stage (URG1) and the beginning of infection (URG2) (Figure 6C, Figure S4C). Among those were genes encoding the F-type and V-type H+- transporting ATPase subunits, as well as the inorganic pyrophosphatase, and ubiquinol-cytochrome c1 reductase subunits. We also observed the upregulation of genes involved in glycolysis (Table S5).

Genes upregulated in URG3 were also clustered into four sub-groups according to correlated gene expression levels for each *Amoebophrya* strain (Table S7), including genes coding for proteins related to Energy metabolism (oxidative phosphorylation), Transport and catabolism (Lysosome) and Signal transduction (MAPK-, Rap1- and Ras- signaling pathways) as found in A120 URG3_3 and A25 URG3_1, respectively (Tables S5, S7).

### Genes putatively implicated in the host-parasite interaction

While the whole-KEGG analyses provided a general survey of the functions enriched during the *Amoebophrya* infection cycle, key functions likely to be implicated in the host-parasitoid interaction were specifically explored using *Plasmodium falciparum* and *Toxoplasma gondii* as references, aiming to secondarily evaluate their expression profiles to determine whether these *Amoebophrya* genes could be involved in such processes.

These well-known human apicomplexan parasites employ different extracellular adhesins for host cell recognition and invasion (Boucher and Bosch, 2015). Some present a combination of trombospondin 1 (TSP1) and von-Willebrand factor type A (vWF) domains. The A120 and A25 transcriptomes code for 45 (A120) and 25 (A25) proteins having at least one TSP1 motif (IPR000884), and 35 (A120) and 20 (A25) proteins having at least one vWF motif (IPR002035). Most of these *Amoebophrya* proteins also have additional domains (Figures S6A–S11A). A focus was done on the five *Amoebophrya* proteins having both TSP1 and vWF domains (5 in A120, 5 in A25) but their domain combinations differ significantly from those found in apicomplexans, with the possible exception of the TRAP-like protein (TLP) (Boucher and Bosch, 2015). The expression profiles of *Amoebophrya* TSP1 and/or vWF coding-genes were distributed in the three URGs. While the five TSP1/vWF-containing proteins were specifically upregulated in URG2 and URG3 in both strains (A120 left and A25 right, Figure S11B) interestingly, proteins with only vWF domains were detected in the URG2 group (6 in A120, Figure S8B, left, and 4 in A25, Figure S8B, right).

Only one apicomplexan rhoptry-like protein (Hakimi et al., 2017) was found in *Amoebophrya*: ROP14 (Table S5). Three and two ROP14-candidate genes were identified in A120 and A25, respectively, each having at least one lipase maturation factor domain (IPR009613) and from 8 to 12 transmembrane segments (Figure S12A). The expression profiles of the two A25 candidate genes and one in A120 (GSA120T00005924001) were bi-modal, with a smal peak at 6 h and a higher one at the end of the infection (24 h), clustering them into the URG3 group (Figure S12B). The two remaining A120 candidates had opposite expression profiles, with GSA120T00016826001 peaking at the beginning (6 h) and late (36 h) infection while GSA120T00002581001 peaked at 24 h (Figure S12B).

As expected for alveolates, several genes encoding members of the alveolin family were identified in the transcriptomes of A120 (5) and A25 (5), with sizes ranging from 285 to 984 aa and containing up to three inner membrane complex (IMCp, IPR022086) protein domains (Figure S13A). Very abundant in *P. falciparum* (12) and *T. gondii* (14), alveolins were therefore found in smaller repertoires in these *Amoebophrya* transcriptomes. Analysis of their expression profiles revealed similar correlated profiles, with increased expressions at beginning and end of the *Amoebophrya* life cycle (Figure S13B).

### Focus on the genes responding to oxidative stress

Reactive oxygen species derive from oxygen reduction, generating a group of highly reactive ions, molecules and radicals. In the host, ROS can be generated as biproducts of the photosynthesis and respiratory chain, participating in biological processes such as the destruction of intracellular pathogens. Host–parasite adaptation dynamics are strongly depending upon environmental changes related to either abiotic or biotic stress factors. In reaction, the parasite develops an oxidative response to protect itself from these stress insults, uprising either from the host or the surrounding environment.

Genes implicated in the oxidative stress response were highly expressed in both parasitic strains. The vast majority of anti-ROS candidate genes (42) are orthologs between both *Amoebophrya* strains, with a slightly higher number of genes found in A120 (Tables S8, S9). Actually, 50 genes (in A120) and 48 genes (in A25) were identified as *Amoebophrya*-ROS counterparts, including those encoding for the ascorbate peroxidase (APX; 6 in both strains), glutaredoxin (GLR; 6 in both strains), monodehydrascorbate reductase (MDAR; 3 in both strains), glutathione peroxidase (GPX; 2 in A120 and 1 in A25), glutathione reductase (GR; 3 in A120 and 2 in A25), peroxiredoxin (PrxR; 4 in both strains), superoxide dismutase (SOD; 2 in A120 and 4 in A25), and thioredoxins (Trx; 24 in A120 and 21 for A25) (Tables S8, S9).

**Figure 7:**
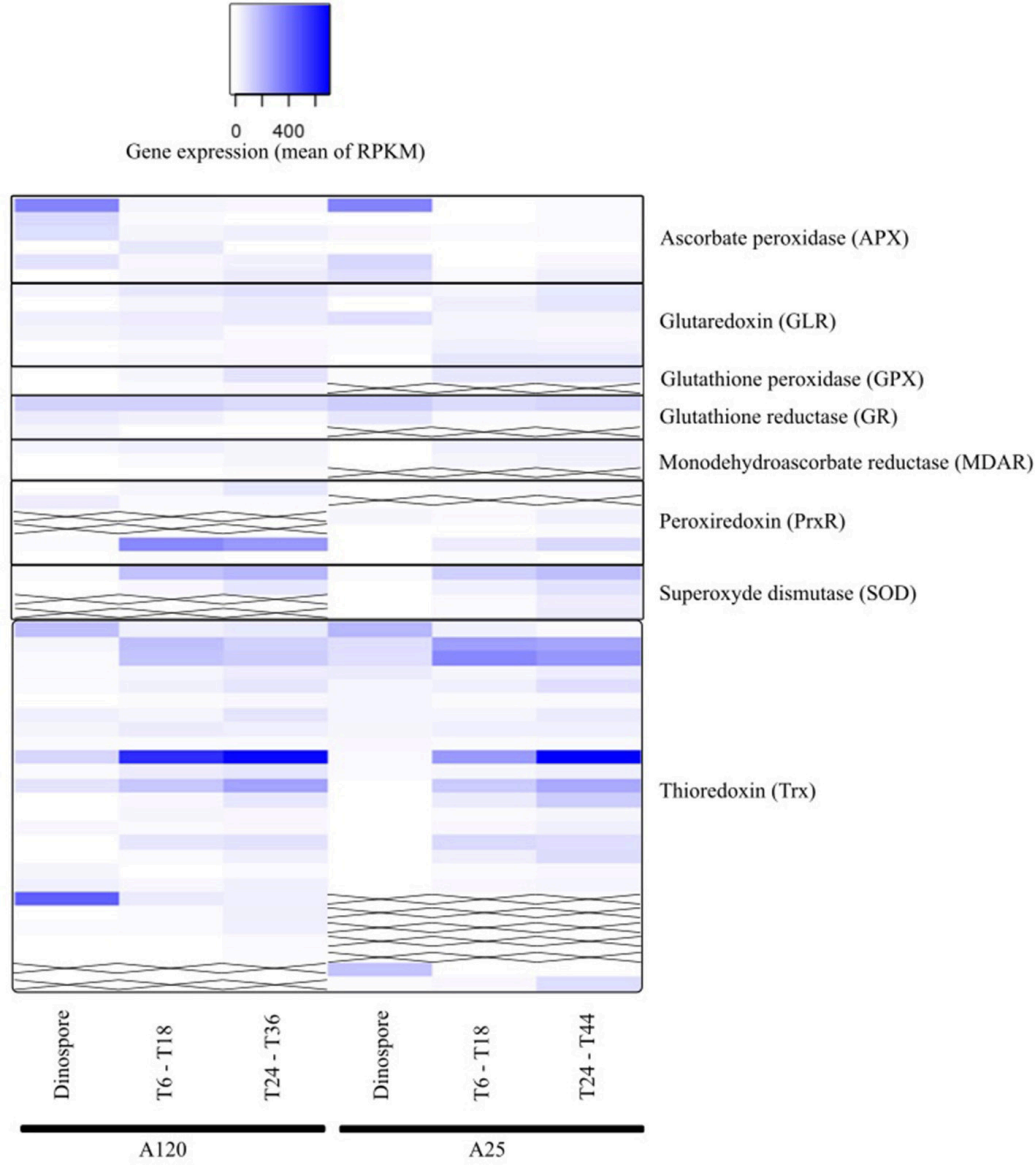
Gene expression of parasite genes possibly involved in anti-ROS responses during *Amoebophrya* infection. The average expression values of anti-ROS genes in *Amoebophrya* A120 **(left)** and A25 **(right)** are represented at the free-living (dinospores), the beginning (T6-T18) and the end (T24-T36) of the host-infection, following a blue-white relative scale. “X” stands for no A120 or A25 counterpart identified.

The anti-ROS *Amoebophrya* candidate genes were found in all three URG groups for both strains (Figure 7, Table S10). Four (A120) and three (A25) APX-like genes were upregulated at the dinospore stage in both strains.

**Figure 8:**
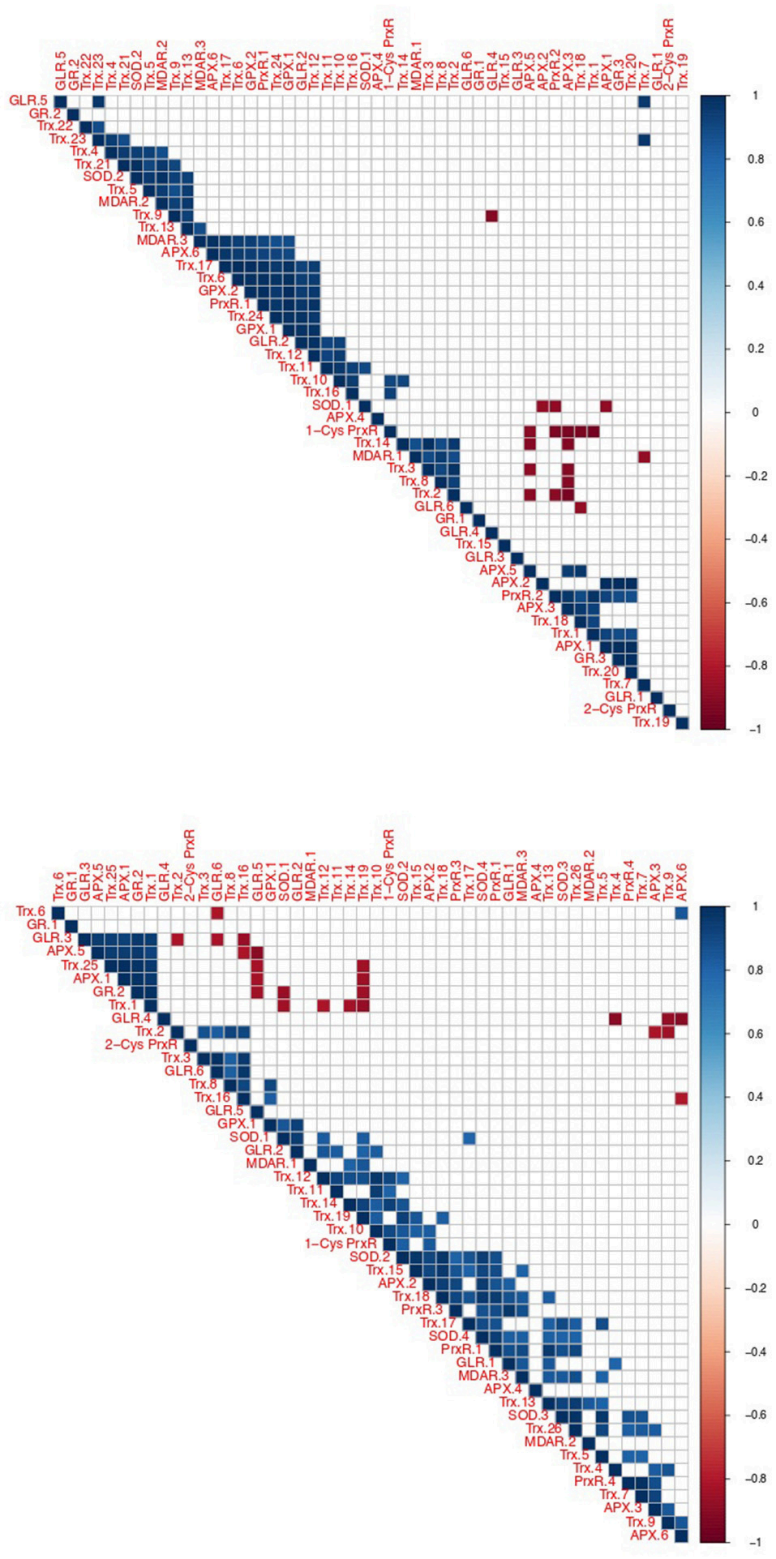
Correlation of oxidative stress response gene expression in *Amoebophrya*. Correlated gene expression of *Amoebophrya* anti-ROS family members. The correlation coefficient for each anti-ROS pair of gene expression values is shown (up, A120; down, A25).

Using correlated gene expression analysis we found putative targets for the *Amoebophrya* anti-ROS system defense. APX genes expression from both strains were found to be correlated with the ones from several anti-ROS effectors, such as members of the PrxR, Trx, or GR families, also upregulated during the free-living stage of the parasite (see Figure 8, Table S3 for details). In A25, APX gene expression, upregulated at dinospore stage, was also strongly correlated with a GLR as to members of the PRx and Trx families (details in Figure 8).

Albeit all these correlated candidate genes were overexpressed during the dinospore stage, most of the anti-ROS genes identified specifically all GLR, two PrxR, all GPX, all SOD and the majority of Trx, were upregulated after the invasion of the host, either at the beginning, the end or both: while URG1 comprises 24 anti-ROS genes (11 for A120 and 13 for A25, respectively), URG2 and URG3 encompass 17 (10 for A120 and 7 for A25), and 52 (25 for A120 and 27 for A25) anti-ROS, respectively.

Interestingly, one A25_APX in URG2 was correlated with two SODs, Trx, PrxR and GLR.1, upregulated at T30. Another one was correlated with PrxR and Trx, with all of them upregulated at T44. While in A120, APX was correlated with MDAR, Trx, GPX and PrxR (upregulated at T24). But no correlation was shown between APX and SOD in A120 (Figure 8, Table S10).

Because of the expression profiles, we focused on APX and SOD sequences in both strains. APX sequences are highly conserved within *Amoebophrya* and *P. marinus* (Figure S14). The phylogeny analyses (Figure S15) showed a high degree of conservation of the APX-related sequences between *Amoebophrya* A120 and A25.

Different forms of SODs can be distinguished depending on their metal cofactors: Cu/Zn, Fe, or Mn. Whereas mostly Fe-SODs were found in several apicomplexan, a conserved Cu/Zn domain was identified in the *Amoebophrya* SOD family (Figures S14, S15, left side). Neither of these SOD-members revealed a significant level of expression during the dinospore stage. Instead, two expression profiles were observed for the SOD-counterparts during the infection process: either at the URG3 or in a bi-modal distribution (max 6 h and 30 h for A120 and 6 h and 24 h for A25, respectively).

## Discussion

Pathogens and parasites rely on their hosts to fuel the resources necessary for their own metabolism and reproduction. As a consequence, many biosynthetic pathways are either lost or highly modified in parasitic species. Over the past years, studies have explored the vital components and essential molecular processes crucial for host-parasite interactions in several apicomplexan model species (Cheeseman and Weitzman, 2015; Swann et al., 2015). A broad number of parasite proteins that play key roles in adjusting the metabolic and signaling pathways of the host cell throughout the infection have been identified (Singh et al., 2009). These proteins allow the parasite to take over the host cellular environment in order to satisfy its own requirements and avoid the host defense mechanisms once inside the host cell. Members of the marine parasitic alveolates belonging to the genus *Amoebophrya* appear to use strategies similar to apicomplexans to invade their hosts (Miller et al., 2012). Here we performed a comparative transcriptomics analysis in order to investigate the host infection processes by two *Amoebophrya* strains, one being a specialist (infecting a single host over the tested strain collection) and the other strain being considered as a more generalist parasite. The initial aim of this approach was to capture the host specialization on the basis of a transcriptomic comparison to pinpoint the lifestyle-specific traits related to the host infection process. This gene expression analysis could thus lead to the identification both of common targets as well as important differences between these *Amoebophrya* infection patterns.

Time-series experiments have been thoroughly used to understand transcriptional changes of apicomplexan parasites during infection (Bozdech et al., 2003; Wastling et al., 2009; Foth et al., 2011; Akbar et al., 2018). Potentially, a time-course comparison between the expression patterns in the overall gene expression could be of great value to explain common features and adaptations favorable for a parasitic lifestyle for *Amoebophrya*. Our analyses were complexified by a relative lack of synchronization between the two experiments, as illustrated by the prevalence of infected hosts (Figure S1). However, we observed significant signal to provide a preliminary understanding of host invasion processes in these two *Amoebophrya* strains by regrouping the parasitoid life cycle into three major steps marked by significant changes in gene expression (Figure 1).

Lu et al. described changes in gene expression of a member of the *Amoebophrya* spp. complex infecting the toxic dinoflagellate host *Alexandrium tamarense* (Lu et al., 2014). RNA-Seq analyses approaches used in this study and Lu's paper were different, making it challenging to compare expression levels among samples. In this sense, our deeper sampling (every 6 h) provides a more refined picture of the transcriptional changes during the infection cycles. Using this approach, functional categories such as Translation and Transcription, emerged as important when the parasite is going through the host cytoplasm to reach its nucleus.

The major differences in gene expression changes took place between the free-living and the infection stages of both parasitoids (Figure 2). For instance, BRITE categories related to transporters such as solute carrier gene families (Höglund et al., 2011), including UDP and nucleoside sugar, metal, and ion (sodium, potassium, and calcium) exchangers, were highly overexpressed during the free-living stage of *Amoebophrya*. Transporters play an essential role in the survival and progression of the life cycle of different apicomplexan parasites (Martin et al., 2009). It is likely that these carriers play an important role in the transport of different charged and uncharged organic molecules as well as inorganic ions across the external cell membrane and throughout the mitochondrial membrane of the parasitoids. This external and internal trafficking between the parasite membranes and the environment would be crucial for the uptake of nutrients and expulsion of waste metabolites.

An abundant range of proteases was as well upregulated during the dinospore stage in *Amoebophrya*. Different assortment of serine, threonine, cysteine, aspartic, and other metalloproteases have been described in protozoan parasites, with several of them involved in the penetration into the host cell, degradation of host nutriments and resistance to the host immune response (Sibley, 2013). For instance, serine proteases from *P. marinus* are active players in extracting nutrients from the oyster host (La Peyre et al., 1995). These proteases may accumulate at this stage in preparation of the future invasion by the dinospore. The overexpression of these proteases, hints to their role for the invasion of the host cell, as observed in *P. marinus* (Joseph et al., 2010).

Very different pathways are required for the life as a parasite inside a host or as a free-living organism. The essential metabolic pathways needed for the survival of the parasite are extremely active during the free-living stage. During this stage, *Amoebophrya* is not only looking for a host to infect but also metabolically active, as shown by the significant overexpression of genes coding for main metabolic pathways, with special emphasis in the ones related with energy production. In fact, most of the gene expression is delegated to the carbohydrate metabolism during dinospore stage, in both strains. Swimming cells require high level of energy for maintaining the motility of the flagella. The nutritional mode of apicomplexan parasites such as *P. falciparum* varies between glycolysis and oxidative phosphorylation depending on their host (Plattner and Soldati-Favre, 2008; Polonais and Soldati-Favre, 2010). In the case of *Amoebophrya*, oxidative phosphorylation seems to be the main resource for energy production needed for dinospores survival whilst glycolysis specifically occurs inside the host, in agreement to Miller (Miller et al., 2012).

Cellobiosidase and endoglucanase activities are known to be used as providers of nutriment resource in the euglenozoan parasite *Leishmania major* (Jacobson and Schlein, 1997). Such activities were as well detected at the dinospore stage in A120 with endoglucanase only in A25. These observations give us a hint on the possible use of such activities once in contact with the host cellulose membrane. Whether cellulose harvested from the host is also a source of nutriment at least in A120 remains to be confirmed.

During this stage of the parasitoid life cycle the parasitoid moves thanks to its flagellum to finding and penetrating their host. Studies have shown the importance of intraflagellar particle transport for both the formation and function of cilium and flagellum (Cao et al., 2010). In both *Amoebophrya* strains, several genes coding for IFT-related proteins were upregulated during the free-living stage in A25. Signaling proteins like MAP kinases and kinesin motor proteins might play a role in the control of flagellum motility in *Trypanosma brucei* (Oberholzer et al., 2011). In addition, kinesin motor proteins were shown as required for its motility (Demonchy et al., 2009). Overexpression of these signaling systems in the swimming dinospores suggests similar roles in *Amoebophrya*.

An outstanding difference in A25-expression levels of genes related to cellular processes was observed when comparing to A120 during the free-living stage of the parasite. In fact, this category is one of the main overrepresented among the upregulated genes in dinospore. These include different tubulin—(e.g., kinesins, dyneins, katanins) and actin—(e.g., myosins and actins) binding proteins involved in the organization of the cytoskeleton. A LRR-containing protein was significantly overexpressed in the dinospore stage of the generalist A120 but not the specialist A25. Among the eukaryotic cytoskeletal proteins, LRR-proteins have already been described as key players for the establishment of infection and pathogen recognition (Kedzierski et al., 2004). Besides, LRR-proteins are also involved in protein-protein interactions and signal transduction (Kobe and Kajava, 2001; Girardin et al., 2002). For instance, the LRR-surface antigen 2 in *Leishmania*, has been implicated in the attachment and invasion of host macrophages (Kedzierski et al., 2004). The overexpression of such LRR-protein in dinospores suggests its possible use in remodeling the parasite cell membrane before the encounter with the host cell as proposed in other apicomplexans.

The beginning of the infection comprises the adhesion to the cell surface and progression of the parasite toward the host nucleus. Besides the protein network needed for the encounter with the host, other specific proteins related to the host degradation would be needed during this stage. Amongst the upregulated genes at the early stages of the infection, we observed overrepresentation of the BRITE categories related to Transcription and Translation, implying that *Amoebophrya* infection resulted in the activation of protein synthesis. Similar pattern of overexpression of proteins involved in the translation process has also been observed in gene expression studies of other apicomplexan parasite models, such as *Toxoplasma* tachyzoites (Cleary et al., 2002), *Cryptosporidium* sporozoites (Lippuner et al., 2018), and *Eimeria* merozoites (Su et al., 2017). The abundance of messenger RNAs for ribosomal proteins, from the early pre-ribosomes, nuclear and cytoplasmic maturation up to the surveillance system (Thomson et al., 2013), recalls extensive protein translation needed after the invasion of the host cell.

Sentrin-specific proteases as well as proteins belonging to the ubiquitination proteolysis pathways were highly overexpressed in the early stages of infection. Protein abundance levels in eukaryotic cells are largely regulated by the ubiquitin system, using complex network of enzymes responsible for the addition and removal of ubiquitin molecules from a protein. Usually related to the proteasome degradation pathway, this ubiquitin system is also involved in several proteasome-independent processes. Host-cell invasion and cyst genesis has been related to the SUMOylation mechanisms in *Toxoplasma* (Braun et al., 2009), as well as in other parasitic protozoa such as *Plasmodium* (Issar et al., 2008) and *Trypanosoma* (Shang et al., 2009). Moreover, modulation of the host SUMOylation machinery have been as well linked to both *Plasmodium* and *Toxoplasma* for efficient infection (Maruthi et al., 2017). Taken together, our data could suggest a SUMO/ubiquitination mechanism not only relates to the invasion process but as a way to transform/assimilate host proteins before the sporont maturation. Later studies in *Plasmodium* have as well shown SUMOylation as an important effector in the oxidative stress response (Reiter et al., 2013). In addition, previous studies have shown the importance of HSP overexpression to overcome environmental stress in other apicomplexan parasites (Vonlaufen et al., 2008). Heat shock proteins (HSP40, HSP60, DNAJ) were as well upregulated during the beginning of *Amoebophrya* infection, indicating their possible role as a parasite defense effector against stress.

During the late infection stage flagellum and other organelles are produced, leading to a multi-nucleated organism made of still-unseparated cells also known as the vermiform stage that emerges from the hollowed host cell (Miller et al., 2012). The vermiform later divides into numerous free-living dinospores that swim in the water column in the search of new host cells to infect (Figure 1). Concomitant upregulation of energy and carbohydrate metabolisms suggests that a high demand for energy is required to form the vermiform and future daughter cells and the vermiform. In parallel, genes related to transport and catabolism BRITE categories, as well as cell growth/death are also upregulated significantly at the final stages of maturation. For instance, genes encoding for proteins likely belonging to the IFT complex and cell division were strongly overexpressed at the end of the infection in both *Amoebophrya* strains (Table S5).

Different invasion strategies were established by the apicomplexan parasites depending on their host (Gubbels and Duraisingh, 2012). Among them, the invasion machineries employed by the well-known *P. falciparum* and *T. gondii* models help the orientation of the parasite and the adhesion. For that, different proteins are used by the parasites: the ones to interact with the host and the ones to invade. A common feature consists in the involvement of multiprotein complexes, interacting with a large array of ligands, each participating in key cellular and physiological steps (e.g., cell adhesion, parasitophorous vacuole formation, migration, homing, pattern formation, and signal transduction).

Many apicomplexan parasite proteins, responsible of the adhesion to the host, contain a combination of TSP1/vWF domains. That is the case of several thrombospondin-related anonymous proteins, like TRAP, MTRAP, CTRP, TRP, and MIC2. As in *T. gondii, P. falciparum* and Chromerids (Templeton and Pain, 2016), numerous proteins with TSP1 and vWF domains were detected in both *Amoebophrya* strains. Proteins that contain either vWF or TSP1 domains are involved in different cell interaction-cascades. Different TSP1/vWF domain combinations were observed in the three URG groups defined during the *Amoebophrya* life cycle. Experimental validation would be interesting to investigate the possible link between the TSP1/vWF domain architecture and specific events such as adhesion to the host cell, homing in the host's nucleus or specific signaling pathways. A striking difference was observed in A120 compared to A25 in terms of the amount of upregulated proteins containing vWF and TSP1 domains. Moreover, a higher number of these vWF-proteins were expressed at dinospore stage in A120. These observations could be related to *Amoebophrya* life-style. Being a generalist, A120 would possibly need an extensive panel of adhesins so to be able to interact with different host species compared to the specialist A25.

Multimembrane vesicles are associated with rhoptries at the host cell entry point in apicomplexans (Håkansson et al., 2001). The electron-dense cell bodies of *Amoebophrya* species seem to function like rhoptries without being part of an apical complex (Miller et al., 2012). Very few rhoptry proteins are conserved between the apicomplexan parasites and the free-living/symbiotic proto-apicomplexan *Vitrella* and *Chromera*, to the notable exception of ROP9 and ROP14 (Janouškovec et al., 2015). ROP14 proteins, which have been first identified in *T. gondii* rhoptries by proteomics (Bradley et al., 2005) have then been proposed to provide a pore in the pasitophorous membrane to facilitate metabolite exchanges between the parasite and its host cell (Laliberté and Carruthers, 2008). ROP14-like proteins, the only apicomplexan rhoptry protein found in *Amoebophrya*, was also identified in *Vitrella, Chromera, Symbiodinium*, and *Perkinsus* (Janouškovec et al., 2015). Consequently, ROP14 might be important to the biology of these organisms. The expression profiles of the 5 ROP14 candidates identified in *Amoebophrya* parasites suggest they play key roles in the establishment of the parasite within the host cell, to be confirmed experimentally.

Alveolins correspond to a family of proteins involved in a diversity of functions depending on the taxa considered (Gould et al., 2008). A large majority of alveolins are molecular components of cortical alveoli, the common structure used as synapomorphy for Alveolata (Gould et al., 2008). While most alveolins are indeed molecular components of cortical alveoli, usually displaying stage specific expressions, some alveolin members have also been associated with basal body complexes or involved in the apicomplexan parasites gliding motility (Anderson-White et al., 2011; Kono et al., 2012; Dubey et al., 2017). Whether these *Amoebophrya* alveolins contribute to the parasite morphogenesis and mechanical strength or play a role in basal bodies structure and regulation will await experimental investigations. Still, all *Amoebophrya* alveolin candidates have expression profiles compatible with both hypothesis—involvement in parasite morphogenesis and/or basal body structure and regulation—since they are expressed in dinospores then at the end of the infection cycle, presumably during progeny assembly.

The study of stress responses in parasitic protozoa is relevant to both pathogenesis and parasite transmission. For instance, the transition from an acute infection into a latent cystic state is triggered by the level of stress on the cosmopolitan *Toxoplasma gondii* parasite (Weiss and Kim, 2000). Studies have stressed the link between low temperatures and salinity to the low prevalence of *P. marinus* infection (Queiroga et al., 2016). Low levels of dissolved oxygen in coastal waters have also being linked to low infection by *P. marinus* (Breitburg et al., 2015). Also, *P. marinus* survives oxidative stress imposed by it host (Anderson, 1999). SOD proteins catalyze the dismutation of O${}_{2}^{-}$ to H_2_O_2_, which in turn is reduced into H_2_O by catalase peroxidase or GPX (Lesser, 2006). Studies have characterized the role of Fe SOD, GPX and APX proteins in *P. marinus* in the defense against oxidative stress during infection of oysters (Wright et al., 2002; Schott et al., 2003; Joseph et al., 2010). Even though protozoans are considered lacking Cu, Zn SODs (Miller et al., 2012) transcriptionally active genes encoding Cu,Zn SODs were recently described in the protozoan ciliate *Tetrahymena thermophila* (Ferro et al., 2015). Here, we also identified Cu-Zn SODs in both *Amoebophrya* strains. In the specialist *Amoebophrya* strain A25 we observed a strong correlation between SOD and APX activities. Thus, the antioxidant process responding to the host in this strain may be similar to that observed in *P. marinus* (Wright et al., 2002; Asojo et al., 2006). On the contrary, no SOD/APX correlation was detected in A120, suggesting the presence of other yet to be identified anti-ROS mechanism.

Taken together, our work presents a comparative analysis, at a transcriptomic level, of two very different *Amoebophrya* strains. Important differences on the gene expression profiles between these *Amoebophrya* gave us a hint on strain-related infection patterns. Remarkably, and perhaps for the first time in dinoflagellates in general, broad changes in gene expression at the transcriptional level are observed over parasitic stages, leading to the conclusion that expression is likely regulated prior the gene transcription. We highlighted several interesting targets potentially involved in the host invasion and the oxidative parasitic stress answer. Further comparative studies using other *Amoebophrya* strains will provide additional clues on the ability of specialized parasites to infect their primary host and adapt to a novel host. The current interpretation of these data is at the beginning stage, the direct links between the functional of certain proteins and gene expressions yet should be established.

## Data availability statement

The *Amoebophrya* A25 and A120 RNA-Seq datasets analyzed for this study will be available through the ENA database using the project ID PRJEB26803.

## Author contributions

SF, LG, BP, and PW conceived and designed the experiments. LG and EB performed the experiments. SF, IF, LG, and BP analyzed the data. LG, CD, and BN contributed reagents, materials, analysis tools. SF, IF, EK, and BP wrote the main manuscript text. All authors reviewed the manuscript.

### Conflict of interest statement

The authors declare that the research was conducted in the absence of any commercial or financial relationships that could be construed as a potential conflict of interest.
