# Supplementary figures and images for: Comparative Time-Scale Gene Expression Analysis Highlights the Infection Processes of Two Amoebophrya Strains
Source: Front Microbiol. 2018 Oct 2;9:2251. doi: 10.3389/fmicb.2018.02251 (PMC6176090; doi:10.3389/fmicb.2018.02251)

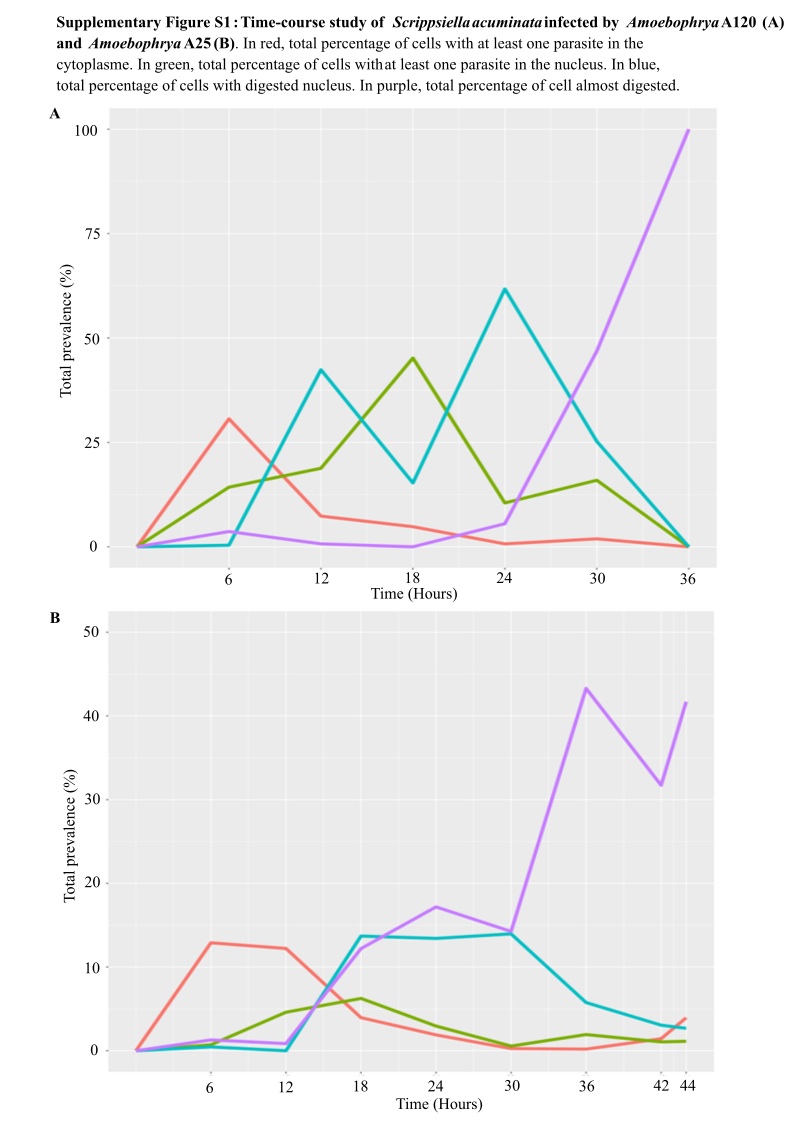

Supplement: Supplementary file 1 [file Image_1.jpeg]

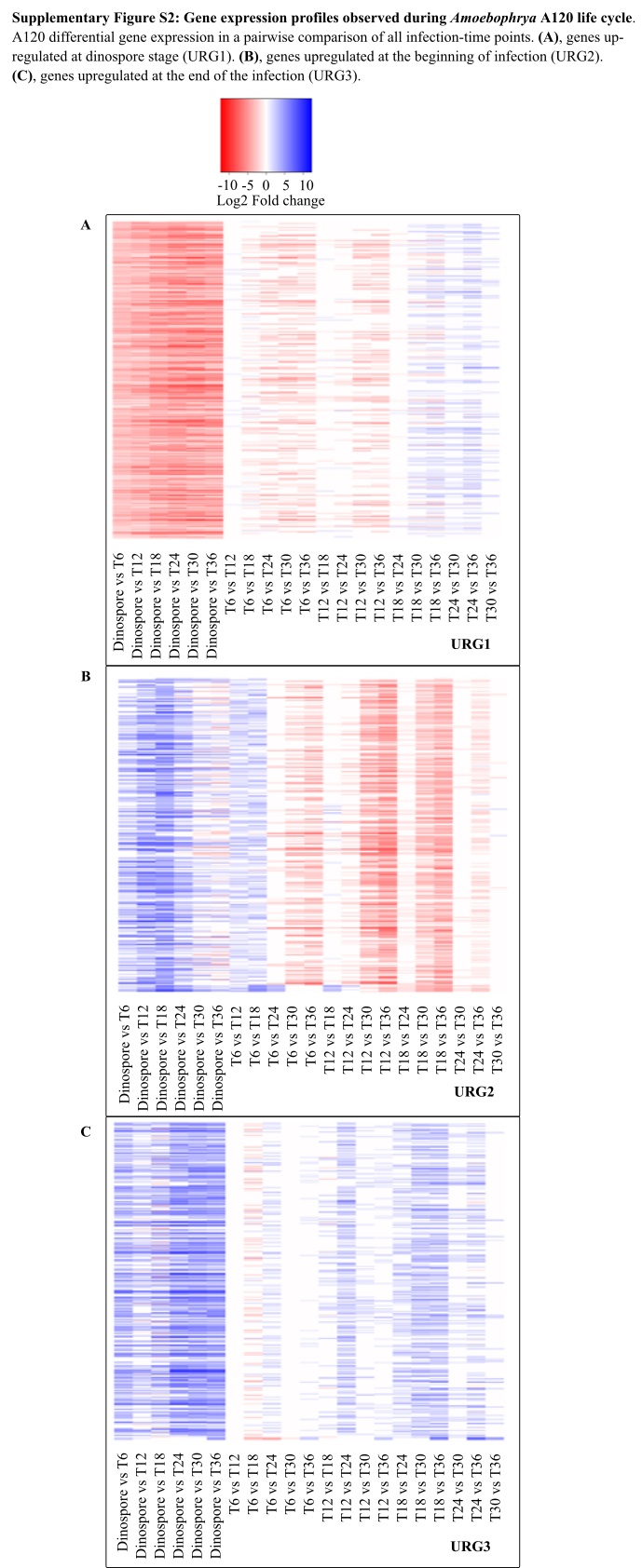

Supplement: Supplementary file 2 [file Image_2.jpeg]

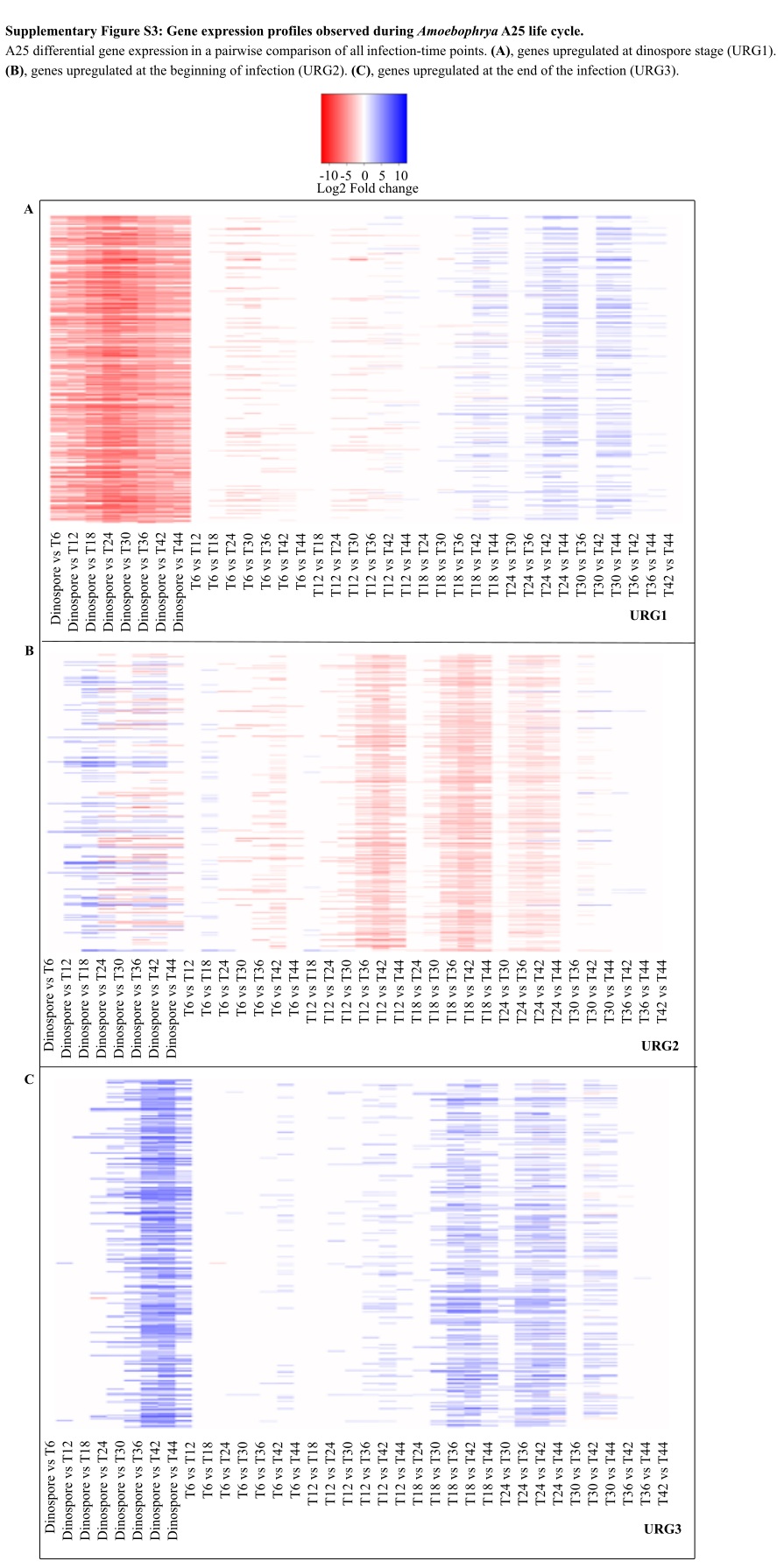

Supplement: Supplementary file 3 [file Image_3.jpeg]

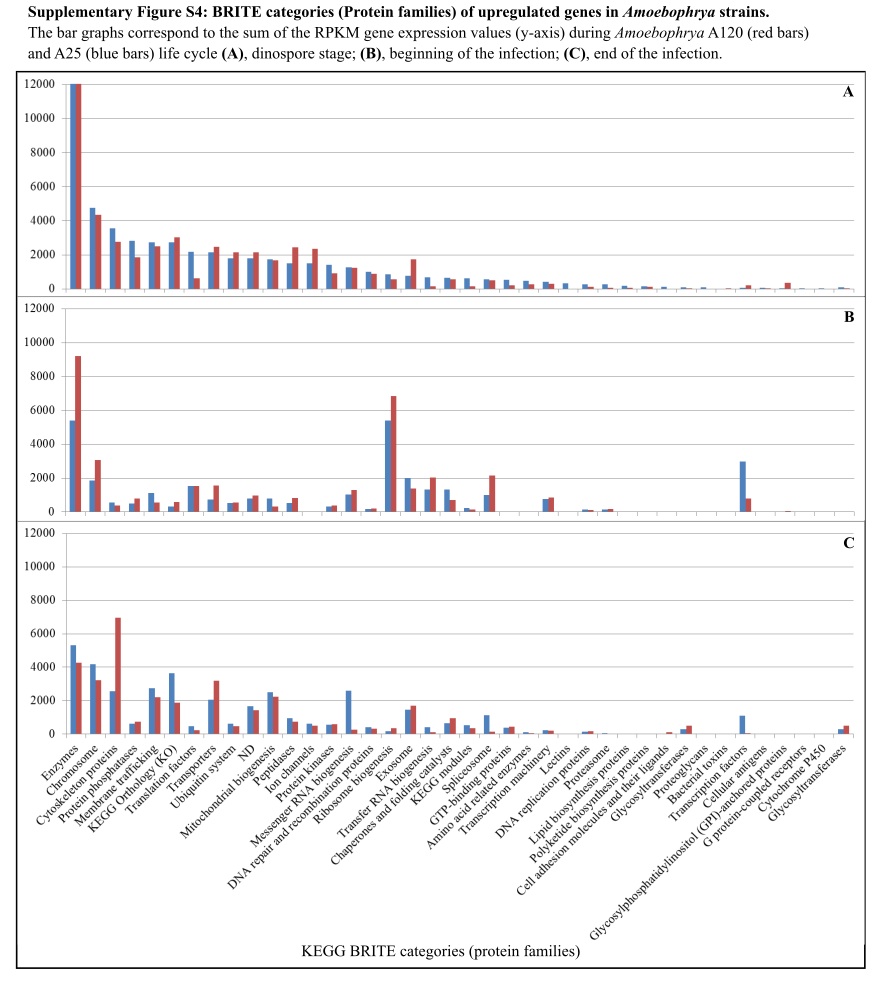

Supplement: Supplementary file 4 [file Image_4.jpeg]

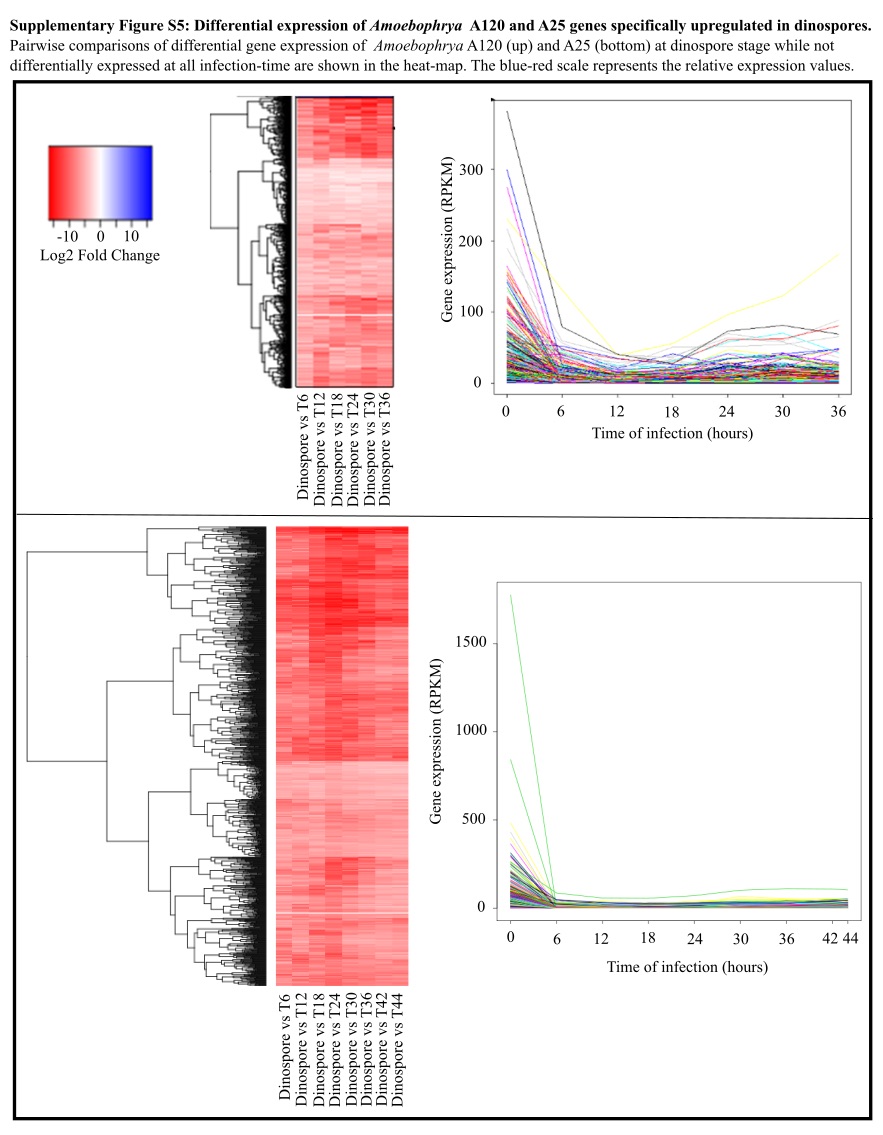

Supplement: Supplementary file 5 [file Image_5.JPEG]

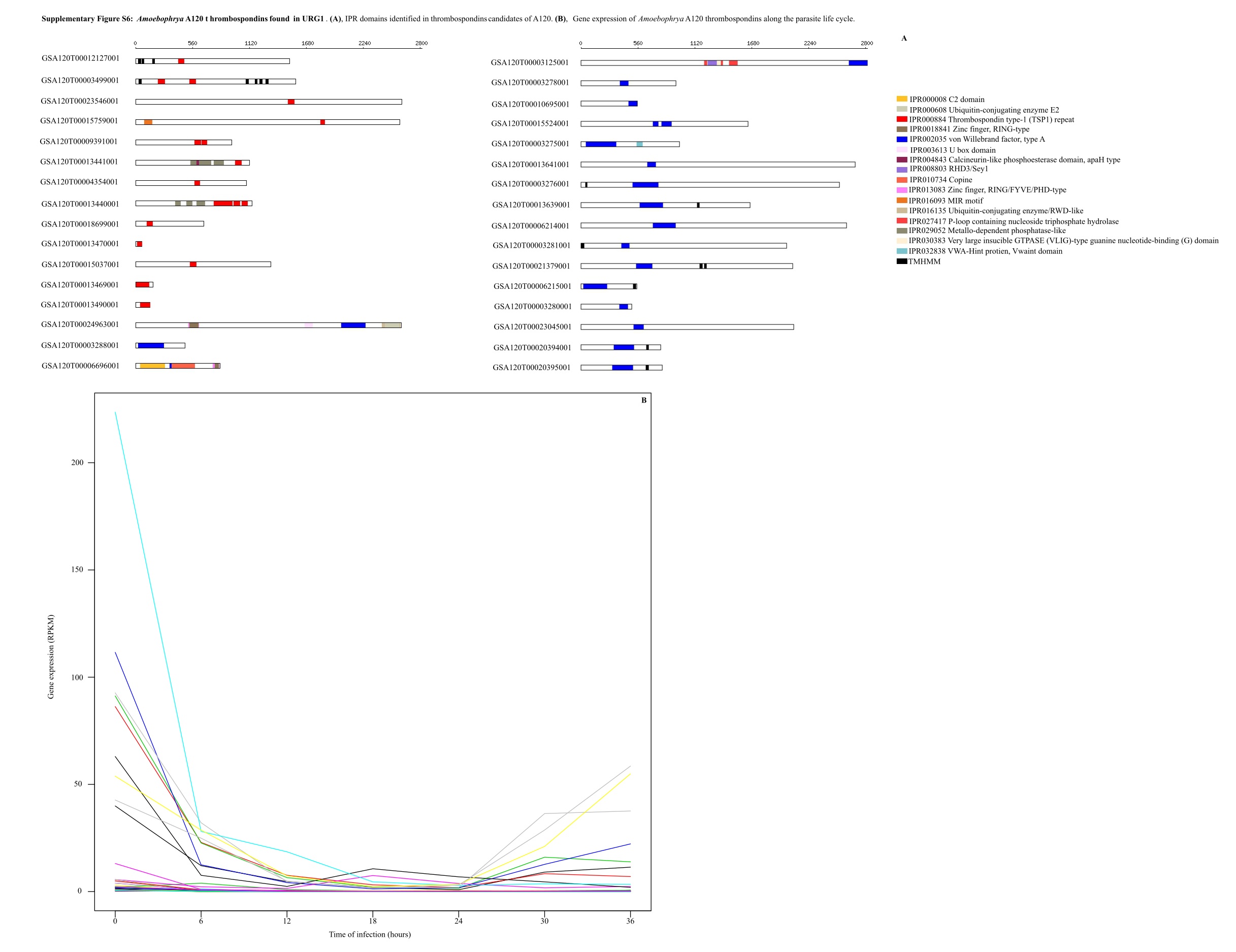

Supplement: Supplementary file 6 [file Image_6.jpeg]

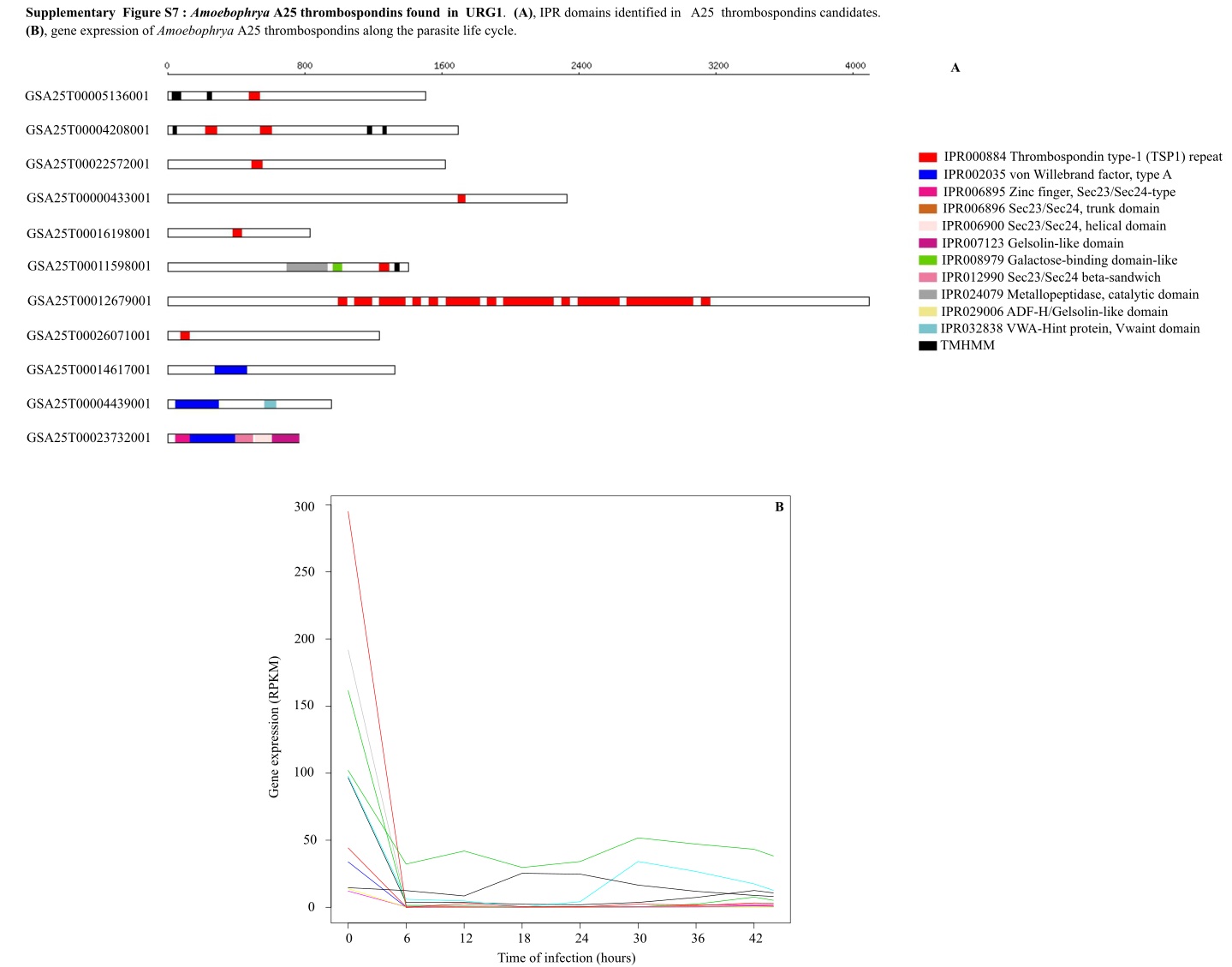

Supplement: Supplementary file 7 [file Image_7.jpeg]

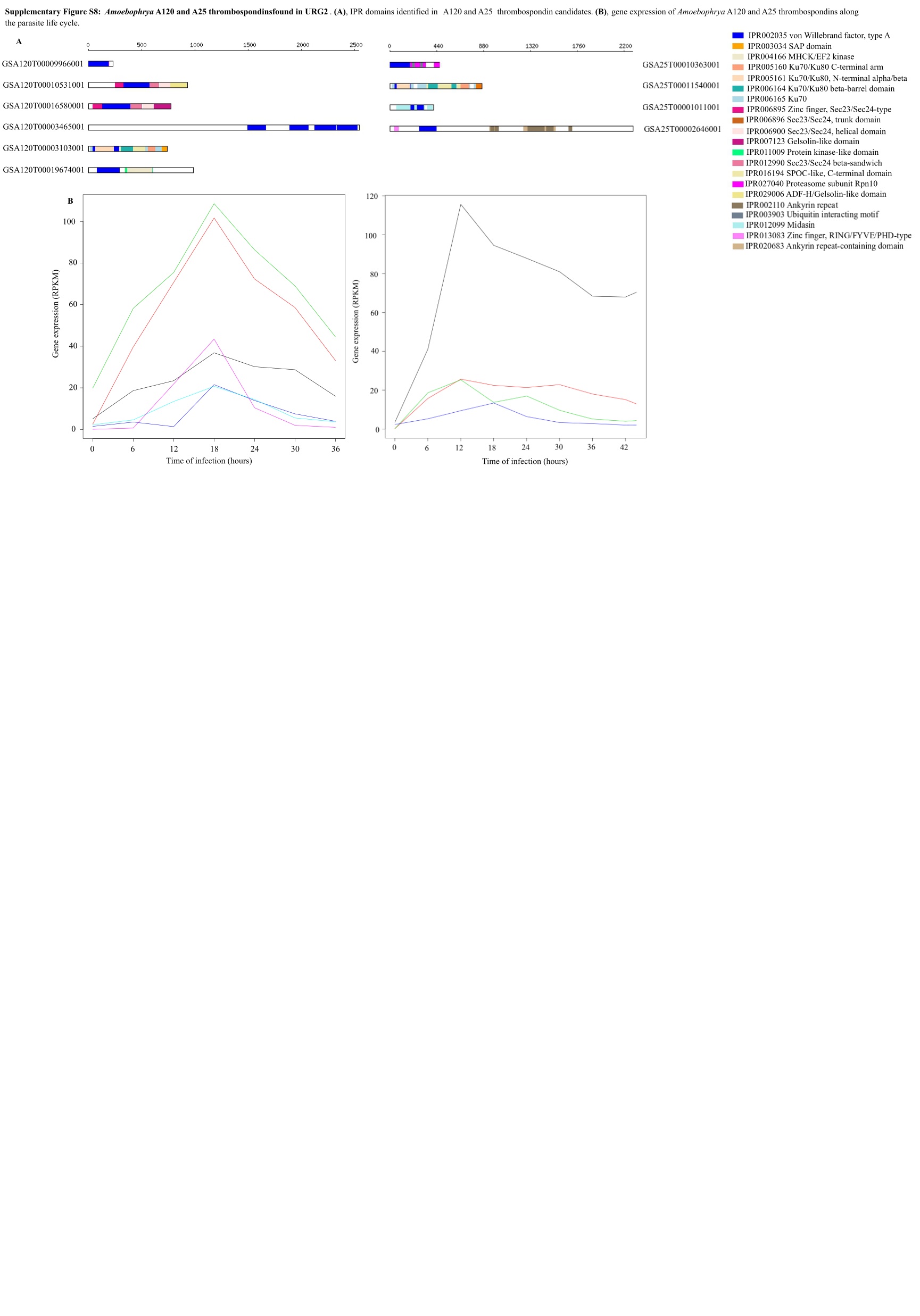

Supplement: Supplementary file 8 [file Image_8.jpeg]

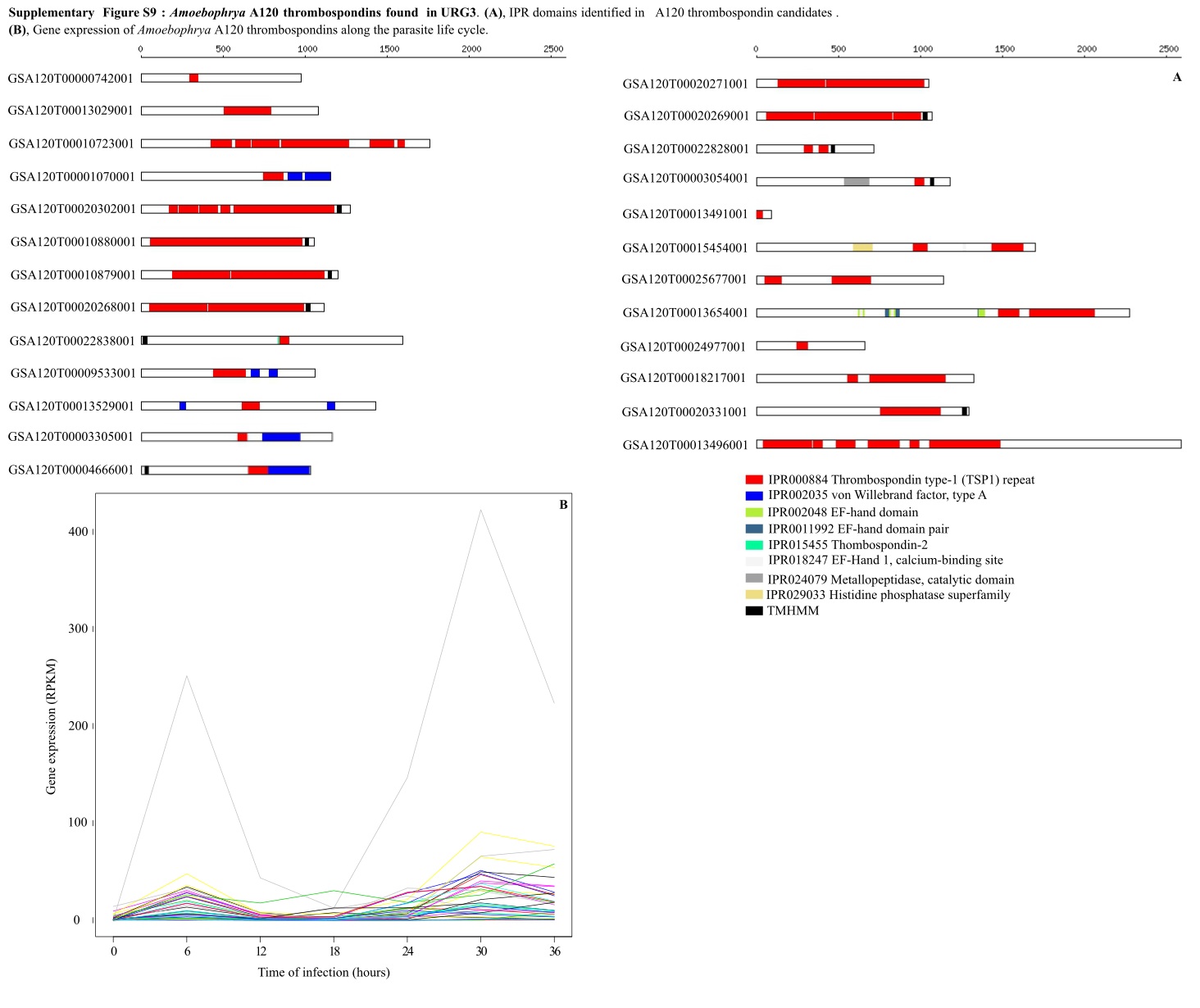

Supplement: Supplementary file 9 [file Image_9.jpeg]

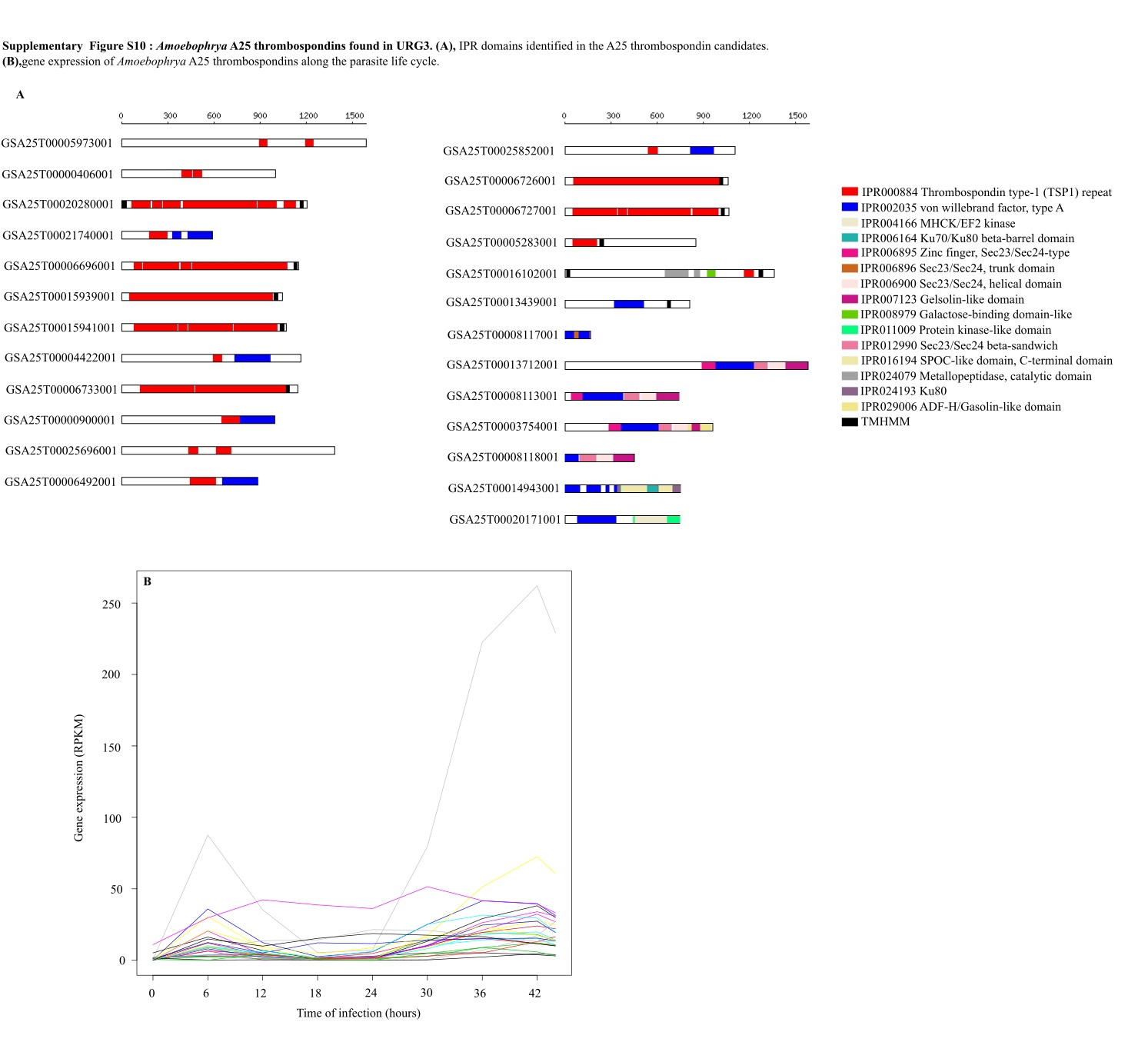

Supplement: Supplementary file 10 [file Image_10.jpeg]

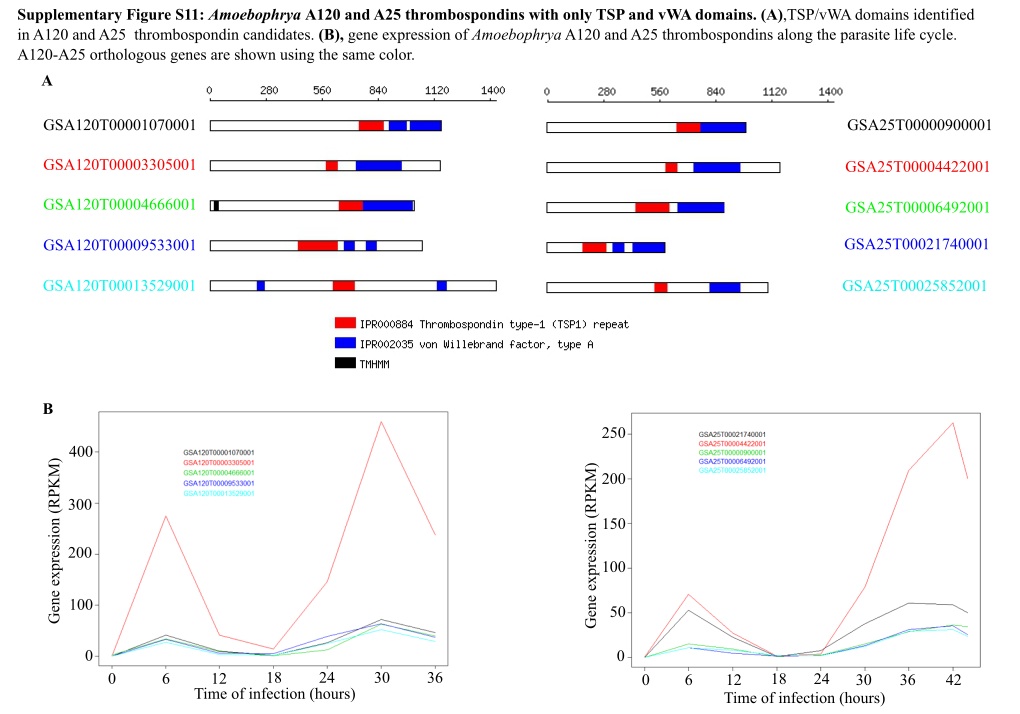

Supplement: Supplementary file 11 [file Image_11.jpeg]

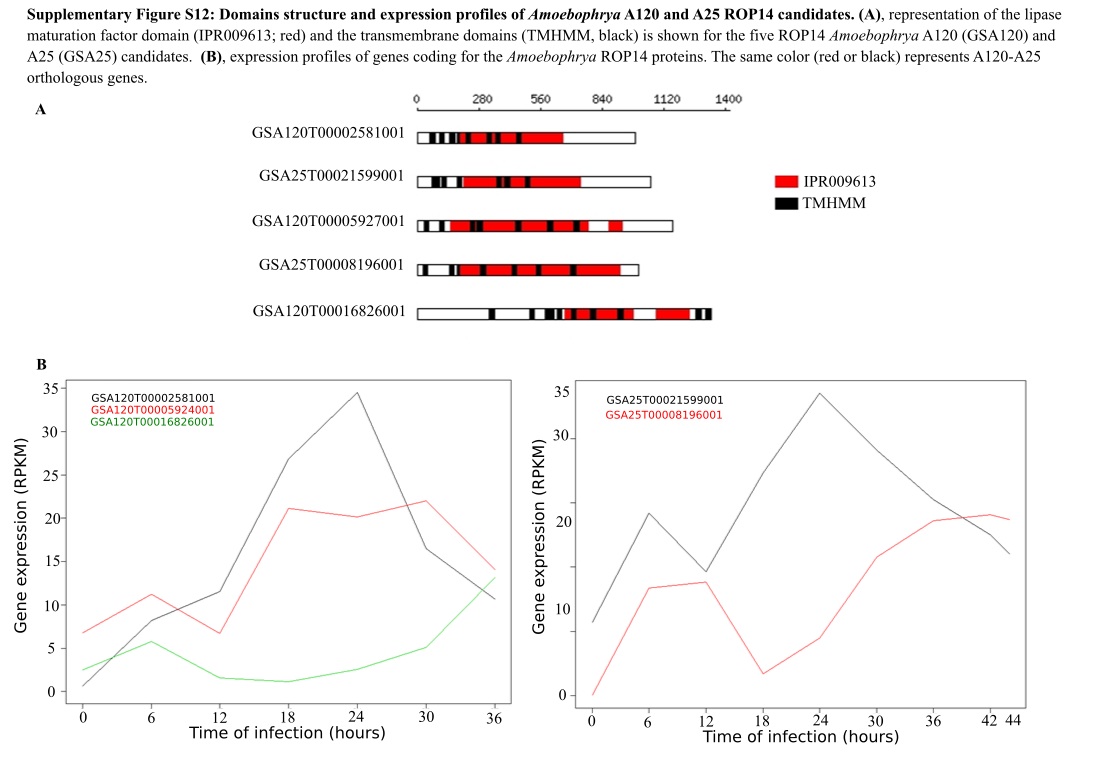

Supplement: Supplementary file 12 [file Image_12.jpeg]

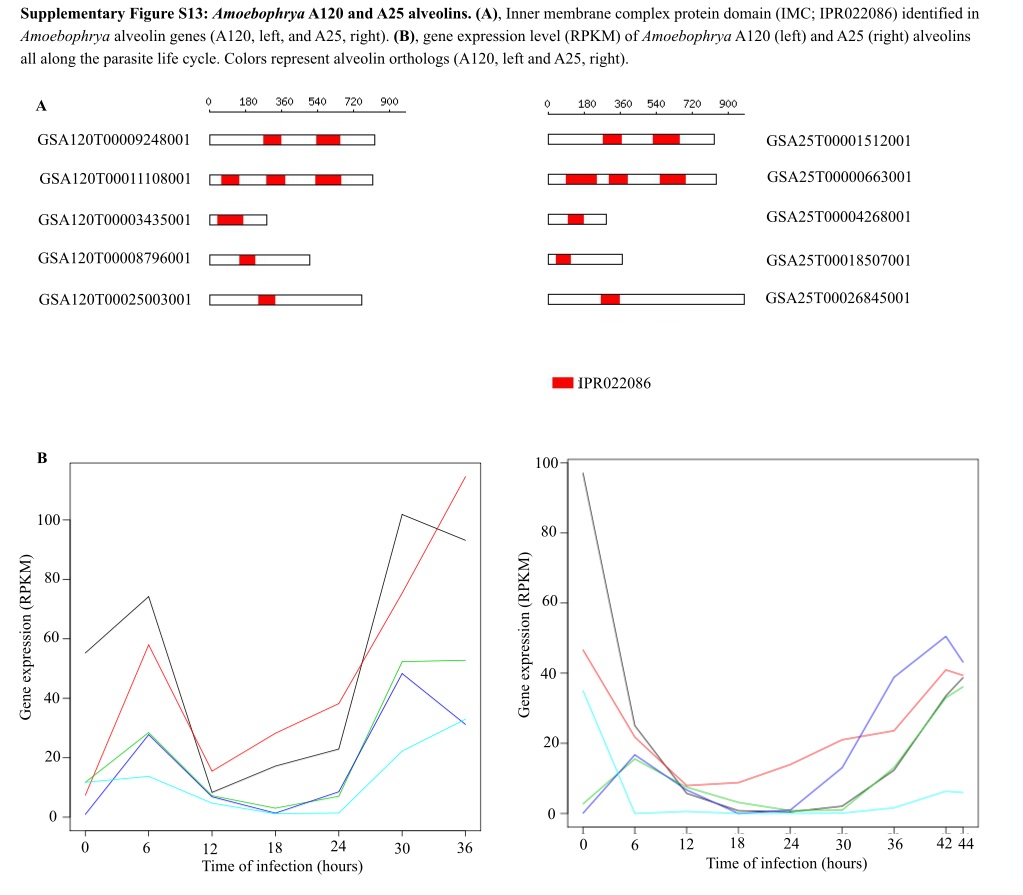

Supplement: Supplementary file 13 [file Image_13.jpeg]

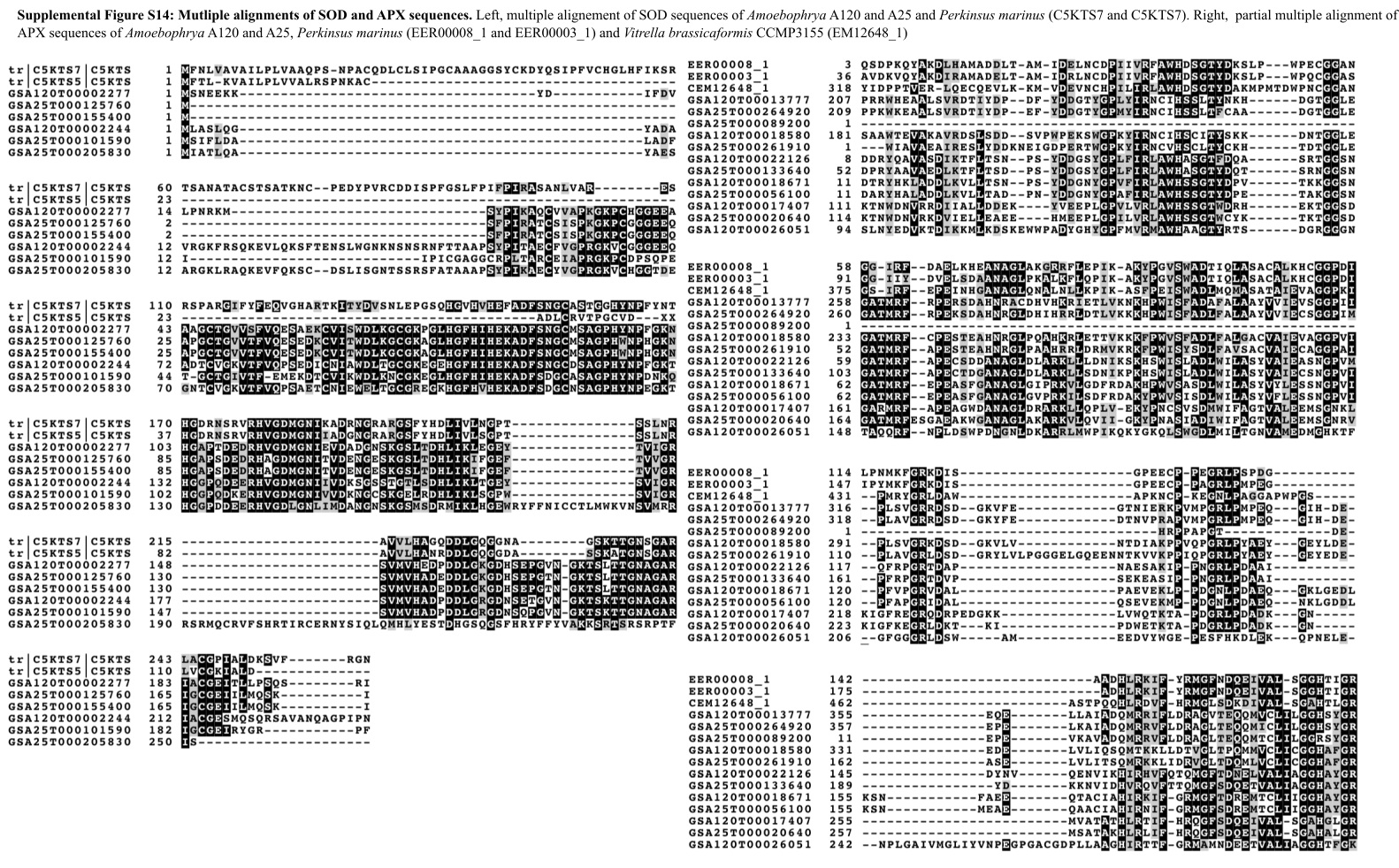

Supplement: Supplementary file 14 [file Image_14.JPEG]

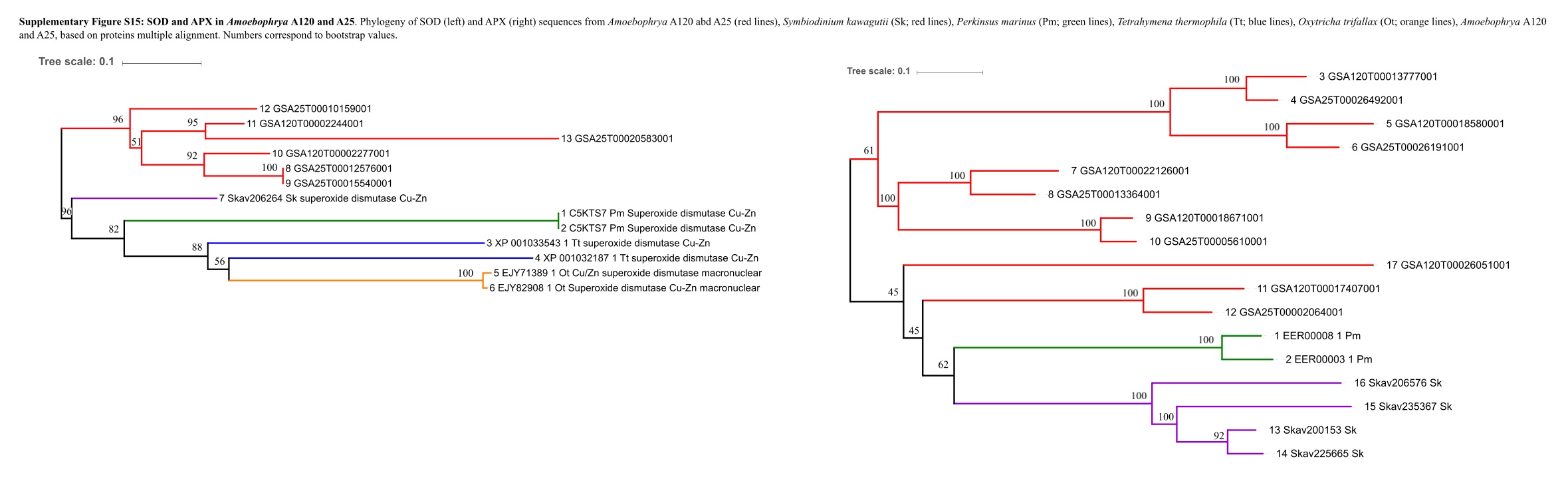

Supplement: Supplementary file 15 [file Image_15.JPEG]
