# Supplementary material for: Comparative Time-Scale Gene Expression Analysis Highlights the Infection Processes of Two Amoebophrya Strains
Source: Front Microbiol. 2018 Oct 2;9:2251. doi: 10.3389/fmicb.2018.02251 (PMC6176090; doi:10.3389/fmicb.2018.02251)
Supplement: Supplementary file 16 [file Table_1.DOCX]

**Supplementary Table S1. *Amoebophrya* A120 and A25 infection dynamics**

**A.** Prevalence of *Amoebophrya* A120 and A25 infection

| Time of infection (hours) | A120 (ST) x ST147  (cell/ml) | A25 (ST) x ST147  (cell/ml) |
| --- | --- | --- |
| 0 | 6567,899863 | 11498,97661 |
| 6 | 4359,765892 | 9283,441558 |
| 12 | 5358,586808 | 9369,801397 |
| 18 | 5058,166249 | 9692,880876 |
| 24 | 5691,653376 | 10279,43058 |
| 30 | 5994,062809 | 9711,619959 |
| 36 | 4678,700539 | 11083,93522 |
| 42 | ND | 9224,56615 |
| 44 | ND | 10502,91448 |

**B.** Host cells harvested during *Amoebophrya* A120 and A25 infection

| Culture | Serie | Time  of infection (hours) | Description | Host (Cells/mL) | Volume collected (mL) | Total host cells harvested |
| --- | --- | --- | --- | --- | --- | --- |
| A120 | A | 0 | T0 only host | 7,40E+03 | 240 | 1,78E+06 |
| A120 | B | 0 | T0 only host | 5,85E+03 | 160 | 9,37E+05 |
| A120 | C | 0 | T0 only host | 6,45E+03 | 200 | 1,29E+06 |
| A120 | A | 6 | A120_T6 | 3,90E+03 | 354,5 | 1,38E+06 |
| A120 | B | 6 | A120_T6 | 4,31E+03 | 354,5 | 1,53E+06 |
| A120 | C | 6 | A120_T6 | 4,87E+03 | 354,5 | 1,73E+06 |
| A120 | A | 12 | A120_T12 | 6,49E+03 | 354,5 | 2,30E+06 |
| A120 | B | 12 | A120_T12 | 4,16E+03 | 354,5 | 1,48E+06 |
| A120 | C | 12 | A120_T12 | 5,42E+03 | 354,5 | 1,92E+06 |
| A120 | A | 18 | A120_T18 | 6,62E+03 | 354,5 | 2,35E+06 |
| A120 | B | 18 | A120_T18 | 4,33E+03 | 354,5 | 1,54E+06 |
| A120 | C | 18 | A120_T18 | 4,23E+03 | 354,5 | 1,50E+06 |
| A120 | A | 24 | A120_T24 | 6,50E+03 | 354,5 | 2,30E+06 |
| A120 | B | 24 | A120_T24 | 5,64E+03 | 354,5 | 2,00E+06 |
| A120 | C | 24 | A120_T24 | 4,94E+03 | 354,5 | 1,75E+06 |
| A120 | A | 30 | A120_T30 | 7,84E+03 | 354,5 | 2,78E+06 |
| A120 | B | 30 | A120_T30 | 5,07E+03 | 354,5 | 1,80E+06 |
| A120 | C | 30 | A120_T30 | 5,07E+03 | 354,5 | 1,80E+06 |
| A120 | A | 36 | A120_T36 | 5,32E+03 | 354,5 | 1,89E+06 |
| A120 | B | 36 | A120_T36 | 3,62E+03 | 354,5 | 1,28E+06 |
| A120 | C | 36 | A120_T36 | 5,10E+03 | 354,5 | 1,81E+06 |
| A25 | A | 0 | T0 only host | 1,36E+04 | 380 | 5,18E+06 |
| A25 | B | 0 | T0 only host | 1,16E+04 | 214 | 2,48E+06 |
| A25 | C | 0 | T0 only host | 9,27E+03 | 238 | 2,21E+06 |
| A25 | A | 6 | A25_T6 | 9,73E+03 | 354,5 | 3,45E+06 |
| A25 | B | 6 | A25_T6 | 9,62E+03 | 354,5 | 3,41E+06 |
| A25 | C | 6 | A25_T6 | 8,50E+03 | 354,5 | 3,01E+06 |
| A25 | A | 12 | A25_T12 | 1,00E+04 | 354,5 | 3,55E+06 |
| A25 | B | 12 | A25_T12 | 9,61E+03 | 354,5 | 3,41E+06 |
| A25 | C | 12 | A25_T12 | 8,48E+03 | 354,5 | 3,01E+06 |
| A25 | A | 18 | A25_T18 | 1,05E+04 | 354,5 | 3,71E+06 |
| A25 | B | 18 | A25_T18 | 1,03E+04 | 354,5 | 3,64E+06 |
| A25 | C | 18 | A25_T18 | 8,36E+03 | 354,5 | 2,96E+06 |
| A25 | A | 24 | A25_T24 | 1,05E+04 | 354,5 | 3,71E+06 |
| A25 | B | 24 | A25_T24 | 1,07E+04 | 354,5 | 3,78E+06 |
| A25 | C | 24 | A25_T24 | 9,72E+03 | 354,5 | 3,45E+06 |
| A25 | A | 30 | A25_T30 | 1,22E+04 | 354,5 | 4,33E+06 |
| A25 | B | 30 | A25_T30 | 8,74E+03 | 354,5 | 3,10E+06 |
| A25 | C | 30 | A25_T30 | 8,18E+03 | 354,5 | 2,90E+06 |
| A25 | A | 36 | A25_T36 | 1,21E+04 | 354,5 | 4,29E+06 |
| A25 | B | 36 | A25_T36 | 1,14E+04 | 354,5 | 4,03E+06 |
| A25 | C | 36 | A25_T36 | 9,78E+03 | 354,5 | 3,47E+06 |
| A25 | A | 42 | A25_T42 | 1,05E+04 | 354,5 | 3,71E+06 |
| A25 | B | 42 | A25_T42 | 8,47E+03 | 354,5 | 3,00E+06 |
| A25 | C | 42 | A25_T42 | 8,75E+03 | 354,5 | 3,10E+06 |
| A25 | A | 44 | A25_T44 | 1,31E+04 | 354,5 | 4,64E+06 |
| A25 | B | 44 | A25_T44 | 1,10E+04 | 354,5 | 3,88E+06 |
| A25 | C | 44 | A25_T44 | 7,46E+03 | 354,5 | 2,65E+06 |
