# Supplementary material for: Comparative Time-Scale Gene Expression Analysis Highlights the Infection Processes of Two Amoebophrya Strains
Source: Front Microbiol. 2018 Oct 2;9:2251. doi: 10.3389/fmicb.2018.02251 (PMC6176090; doi:10.3389/fmicb.2018.02251)
Supplement: Supplementary file 17 [file Table_2.DOCX]

**Supplementary Table S2. Summary of RNA-Seq metrics from *Amoebophrya* A120 and A25 transcriptomes**

| Flowchart step | | A120 | | | A25 | | |
| --- | --- | --- | --- | --- | --- | --- | --- |
| Quality assessment and filtering (samples / reads) | | 24 / 2,408M | | | 29 / 1,889M | | |
| Mapping (samples / reads) | | 24 / 631M | | | 29 / 310M | | |
| Alignment filtering (samples / reads) | | 24 / 424M | | | 29 / 234M | | |
| Samples comparison (samples / reads) | | 16 / 381M | | | 21 / 222M | | |
| Mapped-reads counts (samples / reads) | | 16 /381M | | | 21 / 222M | | |
| Expression profiles clustering | total number of differentially expressed genes | 22,076 | | | 17,193 | | |
|  | number of genes in URGs | URG1 | URG2 | URG3 | URG1 | URG2 | URG3 |
|  |  | 4,349 | 3,543 | 5,287 | 4,737 | 2,540 | 4,490 |
