# Supplementary material for: Comparative Time-Scale Gene Expression Analysis Highlights the Infection Processes of Two Amoebophrya Strains
Source: Front Microbiol. 2018 Oct 2;9:2251. doi: 10.3389/fmicb.2018.02251 (PMC6176090; doi:10.3389/fmicb.2018.02251)
Supplement: Supplementary file 18 [file Table_3.DOCX]

**Supplementary Table S3. RNA-Seq reads metrics on sampled replicates along the *Amoebophrya* A120 (A) and A25 (B) life cycles.** Discarded non-accurate replicates are shown (*). In red, number of hosts reads (*Scrippsiella acuminata*) mapping on *Amoebophrya* genes prediction.

**A**

| **Time of infection (hours)** | **Samples** | **Total number of reads (M)** | **Number of reads mapped on predicted genes (%)** | **Number of reads mapped on predicted genes**  **after filter (%)** |
| --- | --- | --- | --- | --- |
| T0 (host alone) | A | 60 | 2,378,620 (3.98) | 6,103 (0.01) |
|  | B | 57 | 2,256,451 (3.98) | 5,420 (0.01) |
|  | C | 68 | 2,719,360 (4.06) | 6,929 (0.01) |
| Dinospore alone | A | 69 | 62M (90.18) | 59M (85.5) |
|  | B | 73 | 66M (90.23) | 63M (86.3) |
|  | C | 77 | 69M (90.36) | 66M (85.7) |
| T6 | A | 777 | 54,705,662 (7.04) | 8,433,926 (1.09) |
|  | B | 138 | 33,619,368 (24.36) | 1,376,098 (1.01) |
|  | C* | 44 | 2,134,005 (4.90) | 242,350 (0.55) |
| T12 | A | 133 | 16,853,550 (12.7) | 3,473,983 (2.61) |
|  | B | 120 | 7,321,393 (6.11) | 3,418,773 (2.87) |
|  | C* | 54 | 3,089,870 (5.74) | 480,443 (0.89) |
| T18 | A | 104 | 23,077,448(22.19) | 14,155,815 (13.74) |
|  | B | 57 | 11,893,116 (20.85) | 9,472,132 (16.62) |
|  | C* | 124 | 15,783,825 (12.73) | 4,647,398 (3.75) |
| T24 | A* | 42 | 16,186,386 (38.97) | 14,180,363 (33.76) |
|  | B | 43 | 16,815,949 (38.75) | 14,579,436 (33.90) |
|  | C | 50 | 8,788,063 (17.59) | 6,670,774 (13.34) |
| T30 | A* | 47 | 26,852,915 (57.36) | 23,802,505 (50.64) |
|  | B | 54 | 33,295,784 (61.37) | 30,427,944 (56.35) |
|  | C | 53 | 19,397,077 (36.51) | 16,716,521 (31.54) |
| T36 | A | 49 | 28,824,596 (58.94) | 25,774,071 (52.60) |
|  | B | 62 | 39,391,631 (64.07) | 35,752,236 (57.66) |
|  | C | 57 | 26,250,847 (46.05) | 22,816,398 (40.03) |

**B**

| **Time of infection**  **(hours)** | **Samples** | **Total number of reads (M)** | **Number of reads mapped on predicted genes** | **Number of reads mapped on predicted genes**  **after filters** |
| --- | --- | --- | --- | --- |
| T0 (host alone) | A | 61 | 1,466,148 (2.4) | 319 (0.0005) |
|  | B | 43 | 1,272,810 (2.98) | 331 (0.0008) |
|  | C | 41 | 1,145,441 (2.81) | 286 (0.0007) |
| Dinospore alone | A | 73 | 64,854,054 (88.31) | 61,635,018 (84.43) |
|  | B | 72 | 62,551,219 (87.07) | 59,176,362 (82.19) |
| T6 | A* | 42 | 1,536,035 (3.63) | 155,528 (0.37) |
|  | B | 108 | 6,933,423 (6.4) | 812,008 (0.75) |
|  | C | 110 | 6,835,489 (6.2) | 531,292 (0.48) |
| T12 | A* | 52 | 2,065,070 (3.99) | 294,122 (0.57) |
|  | B | 126 | 7,359,910(5.8) | 934,639 (0.74) |
|  | C | 100 | 6,468,248 (6.5) | 1,112,302 (1.11) |
| T18 | A* | 42 | 1,981,300 (4.71) | 648,976 (1.55) |
|  | B | 118 | 9,440,346 (8) | 2,479,994 (2.1) |
|  | C | 127 | 12,353,213 (9,7) | 5,130,849 (4.02) |
| T24 | A | 47 | 3,074,530 (6.53) | 1,679,166(3.58) |
|  | B* | 49 | 3,479,901 (7.17) | 1,968,530 (4.02) |
|  | C | 63 | 6,537,587 (10.45) | 4,643,548 (7.37) |
| T30 | A | 45 | 4,272,467 (9.47) | 2,663,348 (5.92) |
|  | B | 59 | 6,512,574 (11.02) | 4,674,987 (7.92) |
|  | C | 51 | 10,210,088 (20.02) | 8,826,651 (17.31) |
| T36 | A | 51 | 7,597,097 (15.01) | 6,063,025 (11.89) |
|  | B | 58 | 10,264,462 (17.70) | 8,425,149 (14.53) |
|  | C | 49 | 11,427,820 (23.14) | 9,881,339 (20.17) |
| T42 | A | 52 | 8,749,265 (16.92) | 7,231,610 (13.91) |
|  | B | 51 | 10,432,052 (20.60) | 8,877,699 (17.41) |
|  | C | 55 | 17,878,485 (32.47) | 16,033,018 (29.15) |
| T44 | A | 52 | 5,384,433 (10.37) | 3,968,295 (7.63) |
|  | B | 46 | 8,914,602 (19.26) | 7,565,620 (16.45) |
|  | C* | 46 | 9,954,396 (21.65) | 8,541,949 (18.57) |
