# Supplementary material for: Comparative Time-Scale Gene Expression Analysis Highlights the Infection Processes of Two Amoebophrya Strains
Source: Front Microbiol. 2018 Oct 2;9:2251. doi: 10.3389/fmicb.2018.02251 (PMC6176090; doi:10.3389/fmicb.2018.02251)
Supplement: Supplementary file 19 [file Table_4.DOCX]

### Supplementary Table S4. Number of differentially expressed genes comparing pairwise time points of the experiment of *Amoebophrya* A120 (A) and A25 (B) infecting *Scrippsiella acuminata*.

**A**

| All vs all | T6 | T12 | T18 | T24 | T30 | T36 |
| --- | --- | --- | --- | --- | --- | --- |
| T0 | 14,118 | 13,345 | 16,940 | 16,545 | 15,798 | 16,336 |
| T6 |  | 3,153 | 8,323 | 6,283 | 3,256 | 3,902 |
| T12 |  |  | 2,852 | 4,752 | 4,985 | 6,791 |
| T18 |  |  |  | 3,206 | 10,357 | 13,684 |
| T24 |  |  |  |  | 3,646 | 9,079 |
| T30 |  |  |  |  |  | 1,494 |

**B**

| All vs all | T6 | T12 | T18 | T24 | T30 | T36 | T42 | T44 |
| --- | --- | --- | --- | --- | --- | --- | --- | --- |
| T0 | 3,357 | 4,440 | 6,282 | 8,797 | 7,615 | 10,982 | 11,802 | 6,898 |
| T6 |  | 16 | 725 | 1,519 | 901 | 843 | 1,546 | 233 |
| T12 |  |  | 197 | 800 | 793 | 2,379 | 4,324 | 1,900 |
| T18 |  |  |  | 379 | 1,633 | 5,691 | 8,475 | 5,193 |
| T24 |  |  |  |  | 991 | 5,654 | 9,096 | 6,191 |
| T30 |  |  |  |  |  | 408 | 4,978 | 3,094 |
| T36 |  |  |  |  |  |  | 460 | 334 |
| T42 |  |  |  |  |  |  |  | 1 |
