# Supplementary material for: Comparative Time-Scale Gene Expression Analysis Highlights the Infection Processes of Two Amoebophrya Strains
Source: Front Microbiol. 2018 Oct 2;9:2251. doi: 10.3389/fmicb.2018.02251 (PMC6176090; doi:10.3389/fmicb.2018.02251)
Supplement: Supplementary file 21 [file Table_6.DOCX]

### Supplementary Table S6. Correlated gene expression profiles identified in *Amoebophrya* A120 (A) and A25 (B) URG2 groups. “ND” stands for not determined.

**A**

|  | Number of genes | Number of genes with KEGG annotation | Mean of correlation | Median of correlation | Time point of pic of expression | BRITE category most represented |
| --- | --- | --- | --- | --- | --- | --- |
| Group 1 | 1761 | 699 | 0.87 | 0.90 | T12 | Translation |
| Group 2 | 380 | 101 | 0.77 | 0.80 | T18 | Signal transduction |
| Group 3 | 356 | 41 | 0.93 | 0.95 | T6-T12 | Carbohydrate metabolism |
| Group 4 | 175 | 14 | 0.88 | 0.93 | T6 | ND |
| Group 5 | 25 | 6 | 0.74 | 0.78 | T12-T18 | ND |
| Group 6 | 742 | 111 | 0.86 | 0.89 | T18 | Folding, sorting and degradation |
| Group 7 | 104 | 21 | 0.81 | 0.86 | T6 | Carbohydrate metabolism |

**B**

|  | Number of genes | Number of genes with KEGG annotation | Mean of correlation | Median of correlation | Time point of pic of expression | BRITE category most represented |
| --- | --- | --- | --- | --- | --- | --- |
| Group 1 | 1483 | 656 | 0.78 | 0.81 | T12 | Translation |
| Group 2 | 415 | 106 | 0.74 | 0.78 | T18 | Folding, sorting and degradation |
| Group 3 | 18 | 8 | 0.71 | 0.74 | T18 | ND |
| Group 4 | 223 | 33 | 0.75 | 0.80 | T6-T12 | carbohydrate metabolism |
| Group 5 | 95 | 37 | 0.76 | 0.80 | T12 | Translation |
| Group 6 | 51 | 17 | 0.70 | 0.72 | T12 | ND |
| Group 7 | 109 | 10 | 0.72 | 0.76 | T6 | Signal transduction |
| Group 8 | 135 | 6 | 0.82 | 0.87 | T6 | ND |
| Group 9 | 4 | 1 | 0.81 | 0.82 | T6 | ND |
| Group 10 | 7 | 1 | 0.60 | 0.72 | T18 | ND |
