# Supplementary material for: Comparative Time-Scale Gene Expression Analysis Highlights the Infection Processes of Two Amoebophrya Strains
Source: Front Microbiol. 2018 Oct 2;9:2251. doi: 10.3389/fmicb.2018.02251 (PMC6176090; doi:10.3389/fmicb.2018.02251)
Supplement: Supplementary file 22 [file Table_7.DOCX]

### Supplementary Table S7. Correlated gene expression profiles identified in *Amoebophrya* A120 (A) and A25 (B) URG3 groups. “ND” stands for not determined.

**A.**

|  | Number of genes | Number of genes with KEGG annotation | Mean of correlation | Median of correlation | Time point of pic of expression | BRITE category most represented |
| --- | --- | --- | --- | --- | --- | --- |
| Group 1 | 461 | 28 | 0.96 | 0.97 | T36 | Signal transduction |
| Group 2 | 1409 | 313 | 0.92 | 0.95 | T30 | Signal transduction |
| Group 3 | 1931 | 555 | 0.87 | 0.90 | T30 | transport and catabolism |
| Group 4 | 1486 | 383 | 0.94 | 0.96 | T24 | Folding, sorting and degradation |

**B.**

|  | Number of genes | Number of genes with KEGG annotation | Mean of correlation | Median of correlation | Time point of pic of expression | BRITE category most represented |
| --- | --- | --- | --- | --- | --- | --- |
| Group 1 | 2455 | 681 | 0.86 | 0.88 | T36 | Energy metabolism |
| Group 2 | 1300 | 317 | 0.91 | 0.93 | T42-T44 | ND |
| Group 3 | 708 | 238 | 0.79 | 0.84 | T30 | ND |
| Group 4 | 27 | 5 | 0.89 | 0.91 | T24 | Folding, sorting and degradation |
