# Supplementary material for: Comparative Time-Scale Gene Expression Analysis Highlights the Infection Processes of Two Amoebophrya Strains
Source: Front Microbiol. 2018 Oct 2;9:2251. doi: 10.3389/fmicb.2018.02251 (PMC6176090; doi:10.3389/fmicb.2018.02251)
Supplement: Supplementary file 23 [file Table_8.DOCX]

**Supplementary Table S8A. *Amoebophrya* A120 anti-ROS candidate genes.** *Amoebophrya* A120 anti-ROS counterparts were identifed by BLASTp searches onto the NR database.

| anti-ROS id A120 | Best match name | Best match specie | e_value | Annotation |
| --- | --- | --- | --- | --- |
| GSA120T00013777001 | l-ascorbate peroxidase | *Lichtheimia corymbifera* JMRC:FSU:9682 | 1E-40 | Ascorbate peroxidase APX.1 |
| GSA120T00026051001 | catalase/peroxidase HPI | *Caldilinea aerophila* | 0.0 | Ascorbate peroxidase APX.2 |
| GSA120T00022126001 | L-ascorbate peroxidase | *Volvox carteri f. nagariensis* | 5E-76 | Ascorbate peroxidase APX.3 |
| GSA120T00018580001 | Plant ascorbate peroxidase domain-containing protein | *Rozella allomycis* CSF55 | 3E-33 | Ascorbate peroxidase APX.4 |
| GSA120T00018671001 | ascorbate peroxidase | *Sclerotinia borealis F-4128* | 5E-72 | Ascorbate peroxidase APX.5 |
| GSA120T00017407001 | probable ascorbate peroxidase | *Rhynchosporium agropyri* | 4E-93 | Ascorbate peroxidase APX.6 |
| GSA120T00017162001 | PREDICTED: glutaredoxin-1 | *Strongylocentrotus purpuratus* | 1E-24 | Glutaredoxin GLR.1 |
| GSA120T00010648001 | glutaredoxin-like protein | *Pfiesteria piscicida* | 1E-17 | Glutaredoxin GLR.2 |
| GSA120T00021743001 | Grx4 family monothiol glutaredoxin | *Ghiorsea bivora* | 4E-39 | Glutaredoxin GLR.3 |
| GSA120T00010934001 | glutaredoxin-3-like | *Pomacea canaliculata* | 2E-70 | Glutaredoxin GLR.4 |
| GSA120T00017163001 | glutaredoxin, variant | *Aphanomyces astaci* | 1E-17 | Glutaredoxin GLR.5 |
| GSA120T00014524001 | uncharacterized protein TOT_030000449 | *Theileria orientalis strain Shintoku* | 1E-15 | Glutaredoxin GLR.6 |
| GSA120T00005636001 | Hydroperoxy fatty acid reductase gpx1 | *Pirellula sp.* SH-Sr6A | 2E-21 | Glutathione peroxidase GPX.1 |
| GSA120T00000482001 | glutathione peroxidase | *Klebsiella pneumoniae* | 4E-17 | Glutathione peroxidase GPX.2 |
| GSA120T00021828001 | thioredoxin reductase 1, cytoplasmic, putative | *Perkinsus marinus* ATCC 50983 | 0.0 | Glutathione reductase GR.1 |
| GSA120T00021572001 | glutathione-disulfide reductase | filamentous cyanobacterium Phorm 6 | 4E-77 | Glutathione reductase GR.2 |
| GSA120T00023125001 | glutathione-disulfide reductase | *Daedalea quercina* L-15889 | 9E-18 | Glutathione reductase GR.3 |
| GSA120T00003325001 | monodehydroascorbate reductase, chloroplastic/mitochondrial | *Ziziphus jujuba* | 2E-80 | Monodehydroascorbate reductase MDAR.1 |
| GSA120T00009708001 | Apoptosis-inducing factor 3 | *Symbiodinium microadriaticum* | 1E-59 | Monodehydroascorbate reductase MDAR.2 |
| GSA120T00001518001 | unnamed protein product | *Vitrella brassicaformis* CCMP3155 | 2E-81 | Monodehydroascorbate reductase MDAR.3 |
| GSA120T00007668001 | peroxiredoxin | *Pararhodospirillum photometricum* | 1E-15 | Peroxiredoxin PrxR.1 |
| GSA120T00005100001 | Peroxiredoxin | *Paenibacillus sp.* GP183 | 7E-17 | Peroxiredoxin PrxR.2 |
| GSA120T00009282001 | peroxiredoxin | *Dissulfuribacter thermophilus* | 9E-71 | 1-Cys Peroxiredoxin |
| GSA120T00021697001 | peroxiredoxin 6 | *Culex quinquefasciatus* | 7E-14 | 2-Cys Peroxiredoxin |
| GSA120T00002277001 | copper/zinc superoxide dismutase | *Ulva fasciata* | 4E-64 | Superoxyde dismutase SOD.1 |
| GSA120T00002244001 | superoxide dismutase | *Chrysochromulina sp.* CCMP291 | 7E-59 | Superoxyde dismutase SOD.2 |
| GSA120T00010273001 | Protein disulfide-isomerase A3 | *Symbiodinium microadriaticum* | 5E-30 | Thioredoxin Trx.1 |
| GSA120T00005822001 | thioredoxin domain-containing protein | *Hammondia hammondi* | 3E-27 | Thioredoxin Trx.2 |
| GSA120T00024300001 | thioredoxin-like | *Crassostrea virginica* | 1E-36 | Thioredoxin Trx.3 |
| GSA120T00007667001 | thioredoxin domain-containing protein, putative | *Perkinsus marinus* ATCC 50983 | 3E-36 | Thioredoxin Trx.4 |
| GSA120T00007383001 | Thioredoxin domain-containing protein 9 | *Caenorhabditis elegans* | 2E-36 | Thioredoxin Trx.5 |
| GSA120T00000790001 | NADPH thioredoxin reductase | *Fragilariopsis cylindrus* CCMP1102 | 2E-73 | Thioredoxin Trx.6 |
| GSA120T00015953001 | thioredoxin-like protein | *Leucosporidium creatinivorum* | 1E-08 | Thioredoxin Trx.7 |
| GSA120T00018819001 | Thioredoxin, putative | *Perkinsus marinus* ATCC 50983 | 2E-24 | Thioredoxin Trx.8 |
| GSA120T00003676001 | Thioredoxin | *Phytophthora megakarya* | 1E-23 | Thioredoxin Trx.9 |
| GSA120T00021571001 | thioredoxin-like protein | *Syncephalastrum racemosum* | 3E-69 | Thioredoxin Trx.10 |
| GSA120T00017970001 | Thioredoxin | *Aurantiochytrium sp.* FCC1311 | 2E-24 | Thioredoxin Trx.11 |
| GSA120T00008477001 | thioredoxin, putative | *Perkinsus marinus* ATCC 50983 | 2E-149 | Thioredoxin Trx.12 |
| GSA120T00001395001 | thioredoxin, putative | *Perkinsus marinus* ATCC 50983 | 6E-36 | Thioredoxin Trx.13 |
| GSA120T00022450001 | thioredoxin-like protein | *Cystoisospora suis* | 1E-36 | Thioredoxin Trx.14 |
| GSA120T00005920001 | Thioredoxin-like fold | *Pseudocohnilembus persalinus* | 6E-29 | Thioredoxin Trx.15 |
| GSA120T00004045001 | Thioredoxin-like fold | *Pseudocohnilembus persalinus* | 3E-09 | Thioredoxin Trx.16 |
| GSA120T00011983001 | thioredoxin | *Fasciola gigantica* | 2E-17 | Thioredoxin Trx.17 |
| GSA120T00003784001 | thioredoxin | *Epinephelus coioides* | 8E-11 | Thioredoxin Trx.18 |
| GSA120T00014513001 | nucleoredoxin-like protein 2 isoform X1 | *Parasteatoda tepidariorum* | 2E-19 | Thioredoxin Trx.19 |
| GSA120T00017182001 | thioredoxin | *Micromonas commoda* | 2E-110 | Thioredoxin Trx.20 |
| GSA120T00016709001 | protein disulfide isomerase, putative | *Perkinsus marinus* ATCC 50983 | 7E-59 | Thioredoxin Trx.21 |
| GSA120T00008534001 | thioredoxin | *Muribaculaceae bacterium* DSM 100749 | 1E-05 | Thioredoxin Trx.22 |
| GSA120T00008607001 | thioredoxin I | *Neurospora crassa* OR74A | 8E-08 | Thioredoxin Trx.23 |
| GSA120T00022611001 | thioredoxin | *Theileria orientalis* | 7E-16 | Thioredoxin Trx.24 |

**Supplementary Table S8B. *Amoebophrya* A120 APX candidate genes.** *Amoebophrya* A120 APX counterparts were identifed by BLASTp searches onto the Peroxibase database.

|  | Best match | Organism | E-value | Class |
| --- | --- | --- | --- | --- |
| GSA120T00013777001 | CtoCcP03 | Chrysochromulina tobin (sp. CCMP291) | 9E-55 | [Cytochrome C peroxidase](http://peroxibase.toulouse.inra.fr/search/search_type/Class/Cytochrome+C+peroxidase) |
| GSA120T00026051001 | DdaCP01_Ech586 | Dickeya dadantii | 0 | [Catalase peroxidase](http://peroxibase.toulouse.inra.fr/search/search_type/Class/Catalase+peroxidase) |
| GSA120T00022126001 | GprCcP03 | Gonapodya prolifera | 4E-87 | Cytochrome C peroxidase |
| SA120T00018580001 | AnigCcP01 | Ascodesmis nigricans | [4e-40](http://peroxibase.toulouse.inra.fr/tools/do_blast#15230) | Cytochrome C peroxidase |
| GSA120T00018671001 | KpfeCcP01 | Kalaharituber pfeilii (Terfezia pfeilii) | 1E-77 | Cytochrome C peroxidase |
| GSA120T00017407001 | PsuCcP02 | Proteomonas sulcata | 7E-108 | Cytochrome C peroxidase |
