# Supplementary material for: Comparative Time-Scale Gene Expression Analysis Highlights the Infection Processes of Two Amoebophrya Strains
Source: Front Microbiol. 2018 Oct 2;9:2251. doi: 10.3389/fmicb.2018.02251 (PMC6176090; doi:10.3389/fmicb.2018.02251)
Supplement: Supplementary file 24 [file Table_9.DOCX]

**Supplementary Table S9A. *Amoebophrya* A25 anti-ROS candidate genes.** *Amoebophrya* A25 anti-ROS counterparts were identified by BLASTp searches against the NR database.

| anti-ROS id A25 | Best match name | Best match species | e value | Annotation |
| --- | --- | --- | --- | --- |
| GSA25T00026492001 | l-ascorbate peroxidase | *Lichtheimia corymbifera* JMRC:FSU:9682 | 2E-43 | Ascorbate peroxidase APX.1 |
| GSA25T00008920001 | APx2 - Cytosolic Ascorbate Peroxidase | *Zea mays* | 0.002 | Ascorbate peroxidase APX.2 (partial) |
| GSA25T00013364001 | L-ascorbate peroxidase | *Volvox carteri f. nagariensis* | 3E-76 | Ascorbate peroxidase APX.3 |
| GSA25T00026191001 | l-ascorbate peroxidase | *Lichtheimia corymbifera* JMRC:FSU:9682 | 9E-47 | Ascorbate peroxidase APX.4 |
| GSA25T00005610001 | ascorbate peroxidase | *Sclerotinia borealis* F-4128 | 2E-71 | Ascorbate peroxidase APX.5 |
| GSA25T00002064001 | peroxidase | *Meliniomyces variabilis* F | 2E-91 | Ascorbate peroxidase APX.6 |
| GSA25T00025301001 | glutaredoxin-like | *Eurytemora affinis* | 1E-21 | Glutaredoxin GLR.1 |
| GSA25T00007656001 | glutaredoxin-like protein | *Pfiesteria piscicida* | 4E-19 | Glutaredoxin GLR.2 |
| GSA25T00011065001 | monothiol glutaredoxin, Grx4 family | *Acidocella sp.* 20-63-7 | 8E-42 | Glutaredoxin GLR.3 |
| GSA25T00015892001 | glutaredoxin-3-like | *Exaiptasia pallida* | 6E-66 | Glutaredoxin GLR.4 |
| GSA25T00025302001 | glutaredoxin-like | *Eurytemora affinis* | 2E-20 | Glutaredoxin GLR.5 |
| GSA25T00018858001 | hypothetical protein CSUI_002338 | *Cystoisospora suis* | 1E-18 | Glutaredoxin GLR.6 |
| GSA25T00005881001 | glutathione peroxidase | *Trichococcus collinsii* | 3E-10 | Glutathione peroxidase GPX.1 |
| GSA25T00022285001 | thioredoxin reductase 1, cytoplasmic, putative | *Perkinsus marinus* ATCC 50983 | 0.0 | Glutathione reductase GR.1 |
| GSA25T00003956001 | glutathione-disulfide reductase | *Betaproteobacteria bacterium* RIFCSPLOWO2_12_FULL_65_14 | 8E-43 | Glutathione reductase GR.2 |
| GSA25T00002284001 | monodehydroascorbate reductase, chloroplastic/mitochondrial | *Ziziphus jujuba* | 1E-79 | Monodehydroascorbate reductase MDAR.1 |
| GSA25T00022486001 | pyridine nucleotide-disulfide oxidoreductase domain-containing protein | *Toxoplasma gondii* FOU | 1E-27 | Monodehydroascorbate reductase MDAR.2 |
| GSA25T00017292001 | pyridine nucleotide-disulfide oxidoreductase domain-containing protein | *Cystoisospora suis* | 2E-17 | Monodehydroascorbate reductase MDAR.3 |
| GSA25T00007678001 | peroxidoxin 2, putative | *Perkinsus marinus* ATCC 50983 | 2E-95 | Peroxiredoxin PrxR.1 |
| GSA25T00020810001 | peroxiredoxin | *Mesorhizobium sp.* B7 | 0.002 | Peroxiredoxin PrxR.3 |
| GSA25T00008810001 | peroxiredoxin-6 | *Athalia rosae* | 1E-25 | Peroxiredoxin PrxR.4 |
| GSA25T00019478001 | Peroxiredoxin-2, putative | *Perkinsus marinus* ATCC 50983 | 1E-69 | 1-Cys Peroxiredoxin |
| GSA25T00009941001 | peroxiredoxin-6 | *Copidosoma floridanum* | 1E-16 | 2-Cys Peroxiredoxin |
| GSA25T00012576001 | copper/zinc superoxide dismutase | *Ulva fasciata* | 4E-58 | Superoxyde dismutase SOD.1 |
| GSA25T00020583001 | copper/zinc superoxide dismutase | *Ulva fasciata* | 3E-40 | Superoxyde dismutase SOD.2 |
| GSA25T00010159001 | superoxide dismutase | *Chrysochromulina sp.* CCMP291 | 3E-59 | Superoxyde dismutase SOD.3 |
| GSA25T00015540001 | copper/zinc superoxide dismutase | *Ulva fasciata* | 4E-58 | Superoxyde dismutase SOD.4 |
| GSA25T00027576001 | Protein disulfide-isomerase A3 | *Symbiodinium microadriaticum* | 2E-29 | Thioredoxin Trx.1 |
| GSA25T00001540001 | thioredoxin, putative | *Eimeria necatrix* | 3E-32 | Thioredoxin Trx.2 |
| GSA25T00021796001 | thioredoxin | *Lonchura striata domestica* | 3E-35 | Thioredoxin Trx.3 |
| GSA25T00007776001 | thioredoxin domain-containing protein 9-like | *Eurytemora affinis* | 3E-06 | Thioredoxin Trx.4 |
| GSA25T00016873001 | Thioredoxin domain-containing protein 9 | *Caenorhabditis elegans* | 1E-45 | Thioredoxin Trx.5 |
| GSA25T00011146001 | NADPH thioredoxin reductase | *Fragilariopsis cylindrus* CCMP1102 | 5E-66 | Thioredoxin Trx.6 |
| GSA25T00024539001 | thioredoxin dynein outer arm protein | *Plasmopara halstedii* | 4E-15 | Thioredoxin Trx.7 |
| GSA25T00016222001 | Thioredoxin, putative | *Perkinsus marinus* ATCC 50983 | 4E-29 | Thioredoxin Trx.8 |
| GSA25T00025303001 | Thioredoxin | *Phytophthora megakarya* | 3E-15 | Thioredoxin Trx.9 |
| GSA25T00005362001 | protein disulfide isomerase, putative | *Perkinsus marinus* ATCC 50983 | 6E-147 | Thioredoxin Trx.10 |
| GSA25T00007562001 | thioredoxin H-type, putative | *Perkinsus marinus* ATCC 50983 | 1E-11 | Thioredoxin Trx.11 |
| GSA25T00023808001 | thioredoxin, putative | *Perkinsus marinus* ATCC 50983 | 3E-147 | Thioredoxin Trx.12 |
| GSA25T00014689001 | thioredoxin, putative | *Perkinsus marinus* ATCC 50983 | 9E-43 | Thioredoxin Trx.13 |
| GSA25T00009536001 | thioredoxin | *Theileria parva strain Muguga* | 1E-35 | Thioredoxin Trx.14 |
| GSA25T00019015001 | putative thioredoxin | *Toxoplasma gondii p89* | 4E-34 | Thioredoxin Trx.15 |
| GSA25T00016292001 | Thioredoxin-like fold | *Pseudocohnilembus persalinus* | 3E-07 | Thioredoxin Trx.16 |
| GSA25T00026090001 | thioredoxin | *Candidatus Komeilibacteria bacterium* CG10_big_fil_rev_8_21_14_0_10_41_13 | 4E-17 | Thioredoxin Trx.17 |
| GSA25T00017471001 | thioredoxin 2 | *Lasius niger* | 4E-09 | Thioredoxin Trx.18 |
| GSA25T00003583001 | Nucleoredoxin-like protein 2 | *Symbiodinium microadriaticum* | 1E-18 | Thioredoxin Trx.19 |
| GSA25T00027956001 | Ribonucleoside-diphosphate reductase small chain C | *Symbiodinium microadriaticum* | 3E-16 | Thioredoxin Trx.25 |
| GSA25T00015934001 | protein disulfide-isomerase | *Prunus avium* | 9E-06 | Thioredoxin Trx.26 |

**Supplementary Table S9B. *Amoebophrya* A25 APX candidate genes.** *Amoebophrya* A25 APX counterparts were identifed by BLASTp searches onto the Peroxibase database.

|  | Best match | Organism | E-value | Class |
| --- | --- | --- | --- | --- |
| GSA25T00026492001 | CtoCcP03 | Chrysochromulina tobin (sp. CCMP291) | [2e-57](http://peroxibase.toulouse.inra.fr/tools/do_blast#13470) | Cytochrome C peroxidase |
| GSA25T00008920001 | CgrCcP01 | Ochromonas sp. | 3E-07 | Cytochrome C peroxidase |
| GSA25T00013364001 | GprCcP03 | Gonapodya prolifera | 9E-88 | Cytochrome C peroxidase |
| GSA25T00026191001 | CtoCcP03 | Chrysochromulina tobin (sp. CCMP291) | 1E-58 | Cytochrome C peroxidase |
| GSA25T00005610001 | CtheCcP01 | Chaetomium thermophilum | 1E-77 | Cytochrome C peroxidase |
| GSA25T00002064001 | PsuCcP02 | Proteomonas sulcata | 1E-93 | Cytochrome C peroxidase |
