# Supplementary material for: Comparative Time-Scale Gene Expression Analysis Highlights the Infection Processes of Two Amoebophrya Strains
Source: Front Microbiol. 2018 Oct 2;9:2251. doi: 10.3389/fmicb.2018.02251 (PMC6176090; doi:10.3389/fmicb.2018.02251)
Supplement: Supplementary file 25 [file Table_10.DOCX]

### Supplementary Table S10. Expression level (RPKM) of *Amoebophrya* A120 and A25 anti-ROS candidates. MEL (mean expression level in RPKM); “X” stands for not determined

| Gene ID A120 | Gene ID A25 | Predicted annotation | A120 | | | A25 | | |
| --- | --- | --- | --- | --- | --- | --- | --- | --- |
|  |  |  | **Dinospore expression level** | **MEL at beginning of infection** | **MEL at end of infection** | **Dinospore expression level** | **MEL at beginning of infection** | **MEL at end of infection** |
| GSA120T00013777001 | GSA25T00026492001 | Ascorbate peroxidase APX.1 | 331.74 | 23.18 | 17.27 | 334.27 | 0.72 | 3.68 |
| GSA120T00026051001 | GSA25T00008920001 | Ascorbate peroxidase APX.2 | 99.53 | 7.86 | 0.64 | 0.12 | 0.41 | 10.16 |
| GSA120T00022126001 | GSA25T00013364001 | Ascorbate peroxidase APX.3 | 87.23 | 17.68 | 35.51 | 14.46 | 4.97 | 13.17 |
| GSA120T00018580001 | GSA25T00026191001 | Ascorbate peroxidase APX.4 | 0.35 | 54.94 | 2.68 | 0.39 | 0.05 | 0.26 |
| GSA120T00018671001 | GSA25T00005610001 | Ascorbate peroxidase APX.5 | 71.63 | 17.47 | 29.56 | 104.02 | 2.94 | 14.08 |
| GSA120T00017407001 | GSA25T00002064001 | Ascorbate peroxidase APX.6 | 0.47 | 18.45 | 46.51 | 79.60 | 13.18 | 41.43 |
| GSA120T00017162001 | GSA25T00025301001 | Glutaredoxin GLR.1 | 27.80 | 58.20 | 74.02 | 39.04 | 24.53 | 56.30 |
| GSA120T00010648001 | GSA25T00007656001 | Glutaredoxin GLR.2 | 0.10 | 21.94 | 47.78 | 1.03 | 41.81 | 63.92 |
| GSA120T00021743001 | GSA25T00011065001 | Glutaredoxin GLR.3 | 33.90 | 45.48 | 52.01 | 78.31 | 21.23 | 26.99 |
| GSA120T00010934001 | GSA25T00015892001 | Glutaredoxin GLR.4 | 26.12 | 34.44 | 11.17 | 5.01 | 24.26 | 14.40 |
| GSA120T00017163001 | GSA25T00025302001 | Glutaredoxin GLR.5 | 0.81 | 4.64 | 15.04 | 10.18 | 35.30 | 30.62 |
| GSA120T00014524001 | GSA25T00018858001 | Glutaredoxin GLR.6 | 7.10 | 19.78 | 16.65 | 2.70 | 63.02 | 56.60 |
| GSA120T00005636001 | GSA25T00005881001 | Glutathione peroxidase GPX.1 | 0.59 | 18.59 | 66.91 | 5.94 | 56.48 | 66.73 |
| GSA120T00000482001 | X | Glutathione peroxidase GPX.2 | 0.04 | 4.19 | 16.90 | X | X | X |
| GSA120T00021828001 | GSA25T00022285001 | Glutathione reductase GR.1 | 117.68 | 117.97 | 93.23 | 130.08 | 90.63 | 109.10 |
| GSA120T00021572001 | GSA25T00003956001 | Glutathione reductase GR.2 | 48.83 | 30.36 | 10.72 | 57.56 | 7.77 | 5.74 |
| GSA120T00023125001 | X | Glutathione reductase GR.3 | 27.71 | 1.15 | 0.01 | X | X | X |
| GSA120T00003325001 | GSA25T00002284001 | Monodehydroascorbate reductase MDAR.1 | 22.71 | 37.42 | 26.76 | 0.23 | 33.32 | 34.71 |
| GSA120T00009708001 | GSA25T00022486001 | Monodehydroascorbate reductase MDAR.2 | 0.07 | 5.23 | 19.80 | 0.23 | 24.86 | 29.54 |
| GSA120T00001518001 | GSA25T00017292001 | Monodehydroascorbate reductase MDAR.3 | 0.03 | 1.75 | 6.39 | X | X | X |
| GSA120T00007668001 | GSA25T00007678001 | Peroxiredoxin PrxR.1 | 0.27 | 19.55 | 56.34 | 0.03 | 3.99 | 12.16 |
| GSA120T00005100001 | X | Peroxiredoxin PrxR.2 | 45.72 | 7.24 | 12.51 | X | X | X |
| X | GSA25T00020810001 | Peroxiredoxin PrxR.3 | X | X | X | 19.90 | 11.63 | 37.98 |
| X | GSA25T00008810001 | Peroxiredoxin PrxR.4 | X | X | X | 1.51 | 2.15 | 4.82 |
| GSA120T00009282001 | GSA25T00019478001 | 1-Cys Peroxiredoxin | 5.00 | 317.52 | 276.50 | 1.58 | 44.63 | 100.33 |
| GSA120T00021697001 | GSA25T00009941001 | 2-Cys Peroxiredoxin | 0.67 | 6.28 | 3.14 | 0.39 | 3.72 | 2.93 |
| GSA120T00002277001 | GSA25T00012576001 | Superoxyde dismutase SOD.1 | 7.89 | 164.49 | 192.95 | 3.79 | 124.38 | 173.03 |
| GSA120T00002244001 | GSA25T00020583001 | Superoxyde dismutase SOD.2 | 0.31 | 17.30 | 63.18 | 0.17 | 33.74 | 71.94 |
| X | GSA25T00010159001 | Superoxyde dismutase SOD.3 | X | X | X | 0.52 | 13.24 | 42.38 |
| X | GSA25T00015540001 | Superoxyde dismutase SOD.4 | X | X | X | 3.11 | 14.03 | 48.52 |
| GSA120T00010273001 | GSA25T00027576001 | Thioredoxin Trx.1 | 168.82 | 37.56 | 54.76 | 192.48 | 32.57 | 13.28 |
| GSA120T00005822001 | GSA25T00001540001 | Thioredoxin Trx.2 | 22.54 | 167.98 | 121.05 | 81.29 | 262.57 | 239.65 |
| GSA120T00024300001 | GSA25T00021796001 | Thioredoxin Trx.3 | 8.97 | 152.15 | 128.52 | 79.59 | 326.79 | 279.77 |
| GSA120T00007667001 | GSA25T00007776001 | Thioredoxin Trx.4 | 13.84 | 23.79 | 42.44 | 47.68 | 20.40 | 43.97 |
| GSA120T00007383001 | GSA25T00016873001 | Thioredoxin Trx.5 | 6.70 | 28.60 | 65.34 | 22.61 | 33.33 | 82.35 |
| GSA120T00000790001 | GSA25T00011146001 | Thioredoxin Trx.6 | 0.06 | 5.59 | 14.83 | 21.37 | 5.85 | 11.75 |
| GSA120T00015953001 | GSA25T00024539001 | Thioredoxin Trx.7 | 32.14 | 21.38 | 68.63 | 21.20 | 22.90 | 50.52 |
| GSA120T00018819001 | GSA25T00016222001 | Thioredoxin Trx.8 | 25.33 | 46.27 | 35.94 | 18.48 | 37.14 | 36.70 |
| GSA120T00003676001 | GSA25T00025303001 | Thioredoxin Trx.9 | 5.52 | 4.23 | 10.28 | 16.95 | 8.86 | 13.64 |
| GSA120T00021571001 | GSA25T00005362001 | Thioredoxin Trx.10 | 104.21 | 574.39 | 678.53 | 13.83 | 271.51 | 703.85 |
| GSA120T00017970001 | GSA25T00007562001 | Thioredoxin Trx.11 | 4.30 | 47.16 | 64.80 | 10.80 | 20.58 | 39.74 |
| GSA120T00008477001 | GSA25T00023808001 | Thioredoxin Trx.12 | 68.00 | 150.17 | 246.63 | 2.86 | 137.84 | 230.13 |
| GSA120T00001395001 | GSA25T00014689001 | Thioredoxin Trx.13 | 0.52 | 15.23 | 57.43 | 1.24 | 49.75 | 137.17 |
| GSA120T00022450001 | GSA25T00009536001 | Thioredoxin Trx.14 | 0.26 | 18.75 | 16.00 | 0.58 | 12.81 | 19.34 |
| GSA120T00005920001 | GSA25T00019015001 | Thioredoxin Trx.15 | 14.39 | 9.65 | 17.19 | 0.43 | 15.71 | 35.55 |
| GSA120T00004045001 | GSA25T00016292001 | Thioredoxin Trx.16 | 1.78 | 62.31 | 73.12 | 0.25 | 98.45 | 83.45 |
| GSA120T00011983001 | GSA25T00026090001 | Thioredoxin Trx.17 | 0.57 | 12.43 | 38.05 | 0.20 | 41.04 | 89.21 |
| GSA120T00003784001 | GSA25T00017471001 | Thioredoxin Trx.18 | 20.04 | 2.47 | 7.20 | 0.16 | 4.22 | 15.80 |
| GSA120T00014513001 | GSA25T00003583001 | Thioredoxin Trx.19 | 36.10 | 14.68 | 31.95 | 0.12 | 15.11 | 21.90 |
| GSA120T00017182001 | X | Thioredoxin Trx.20 | 438.44 | 50.49 | 36.64 | X | X | X |
| GSA120T00016709001 | X | Thioredoxin Trx.21 | 3.35 | 11.95 | 41.84 | X | X | X |
| GSA120T00008534001 | X | Thioredoxin Trx.22 | 5.68 | 12.17 | 38.19 | X | X | X |
| GSA120T00008607001 | X | Thioredoxin Trx.23 | 0.18 | 3.31 | 13.24 | X | X | X |
| GSA120T00022611001 | X | Thioredoxin Trx.24 | 0.42 | 1.90 | 3.95 | X | X | X |
| X | GSA25T00027956001 | Thioredoxin Trx.25 | X | X | X | 161.13 | 0.50 | 3.12 |
| X | GSA25T00015934001 | Thioredoxin Trx.26 | X | X | X | 4.44 | 17.45 | 87.40 |
